# Evaluating syphilis transmission among MSM population: a mathematical model for reinfection, two-stage treatment, and treatment failure

**DOI:** 10.3389/fpubh.2026.1841745

**Published:** 2026-06-24

**Authors:** Chidozie W. Chukwu, Dipo Aldila, Maruf A. Lawal, George Obaido

**Affiliations:** 1 Department of Mathematical Sciences, Georgia Southern University, Statesboro, GA, United States; 2 Department of Mathematics, Faculty of Mathematics and Natural Sciences, Universitas Indonesia, Depok, Indonesia; 3 Innovative Mathematics and Predictive Analytics for Complex System and Technology Laboratory (IMPACT Lab), Universitas Indonesia, Depok, Indonesia; 4 Department of Mathematics, University of Tennessee, Knoxville, TN, United States; 5 College of Computing, Data Science and Society, University of California, Berkeley, Berkeley, CA, United States

**Keywords:** joinpoint regression, numerical simulations, reproduction number, sensitivity analysis, treatment

## Abstract

This study develops and analyzes a data-driven compartmental model for the transmission dynamics of syphilis among men who have sex with men (MSM) in the United States, incorporating reinfection, two-stage treatment, treatment failure and behavioral adaptation. The model comprises five epidemiological compartments with parameters that capture temporary immunity, adaptive behavior, and heterogeneous treatment outcomes. Analytical results include the derivation of the basic reproduction number ($R_{0}$), stability analysis of equilibria, and conditions for backward bifurcation, revealing potential disease persistence even when $R_{0}<1.$ Using U.S. surveillance data from 2011 to 2023, joinpoint regression identified three major inflection periods 2017, 2019, and 2021 corresponding to shifts in epidemic trajectories. Model calibration demonstrated strong agreement with reported data, yielding MAPE = 14.41% and RMSE = 9376.66, thereby validating the model's predictive accuracy. Simulation and sensitivity analyses underscore the influence of reinfection, behavioral adaptation, and treatment efficacy on disease persistence. Decision-analytic evaluation indicates that a combined approach of enhanced screening, timely treatment, and doxycycline post-exposure prophylaxis can substantially reduce the syphilis burden. The proposed framework provides a robust, quantitative decision-support tool for optimizing intervention strategies among high-risk MSM populations.

## 1 Introduction

Syphilis remains a persistent public health concern in the United States despite advances in prevention, diagnosis, and treatment. In 2023, more than 209,000 cases were reported nationally, alongside over 2.4 million notifiable sexually transmitted infections (STIs), including 3,882 congenital syphilis cases (1). Although primary and secondary syphilis declined for the first time in more than two decades, decreasing by 10% overall and 13% among gay, bisexual, and other men who have sex with men (MSM) (2).

Syphilis is also a global public health concern, with rising incidence reported across North America, South America, Asia, and Europe. Recent reports from the World Health Organization (WHO) highlight a resurgence in both high- and middle-income countries, driven by behavioral, social, and healthcare access factors (3, 4). In Brazil, the national “Sífilis Não” (Syphilis No) Project has established a large-scale collaborative network focused on surveillance, modeling, and intervention strategies, including the use of interrupted time series analysis to evaluate epidemic trends and public health responses (5). Similarly, studies from China and other regions have advanced understanding of syphilis transmission through mathematical and network-based modeling approaches (6). Collectively, these efforts underscore that syphilis is a widespread and evolving global health challenge. Motivated by this context, our study focuses on MSM populations in the United States while contributing to the broader effort to understand and control syphilis transmission through mathematical modeling.

MSM continues to account for a disproportionate share of incident infections. As such, gains appear fragile, and sustained control remains challenging. Syphilis, caused by *Treponema pallidum*, progresses through primary, secondary, latent, and tertiary stages if untreated. Benzathine penicillin G (BPG) remains the standard therapy, with a single dose recommended for early infection and three weekly doses for late latent or unknown duration (7, 8). Although treatment efficacy is high, reinfection is common in sexual networks with high partner turnover. Additionally, a subset of patients experience a suboptimal serologic response even after guideline-concordant therapy, creating uncertainty regarding treatment failure, the need for re-treatment, and the interpretation of post-treatment serologic trajectories (9, 10). These clinical and epidemiologic complexities highlight the importance of better understanding reinfection risk and treatment response in MSM populations.

Behavioral and structural changes over the past decade have further influenced syphilis transmission. Increasing partner concurrency, the widespread use of geosocial networking applications, and evolving condom practices particularly in the context of HIV pre-exposure prophylaxis (PrEP) have contributed to continued disease spread (11–14). Although PrEP has transformed HIV prevention, debate continues regarding its indirect effects on bacterial STIs through behavioral risk compensation. More recently, doxy-PEP has emerged as a promising strategy to reduce Syphilis and Chlamydia among high-risk MSM and transgender women, leading to targeted clinical guidance (15, 16). However, the long-term population-level impact of doxy-PEP, its potential interaction with screening and partner services, and implications for antimicrobial stewardship remain uncertain.

Mathematical and computational models provide valuable tools for integrating natural history, behavioral dynamics, and intervention mechanisms to inform syphilis control. Prior work has employed deterministic compartmental models, network-based simulations, and agent-based approaches to investigate stage-specific transmission, reinfection, loss of immunity, and intervention strategies (17–23). U.S.-focused analyses have evaluated screening coverage and frequency, partner services, cost-effectiveness, and synergies with HIV prevention (24, 25). Recent studies have explored doxy-PEP implementation, assessing both its potential to reduce incidence and the tradeoffs associated with broad versus targeted deployment (26–28). Work examining multi-pathogen contexts and core-group dynamics emphasizes how concentrated transmission networks and reinfection cycles complicate sustained control (29–31). Empirical findings further reinforce the need to address reinfection and heterogeneous treatment outcomes. For example, Luo et al. (51) reported that secondary syphilis, HIV-positive status, and inconsistent condom use were associated with higher odds of serologic failure or reinfection, underscoring the interplay between clinical and behavioral determinants. Despite these advances, significant knowledge gaps remain. Few models calibrated to U.S. MSM populations explicitly incorporate (i) reinfection stemming from waning or incomplete immunity; (ii) treatment failure and heterogeneous serologic response; (iii) biomedical prevention strategies such as doxy-PEP; and (iv) behavioral changes that could undermine intervention effectiveness. These limitations make it difficult to compare alternative intervention portfolios and to identify conditions under which control might be fragile. See other literature on infectious diseases (32, 33).

To address these needs, we develop a compartmental model tailored to U.S. MSM that integrates clinical staging, reinfection, heterogeneous response to treatment, and behavior-change mechanisms that influence transmission. The model represents two-stage BPG therapy consistent with clinical practice and allows for treatment non-response through reduced treatment efficacy and variable serologic decay. We evaluate a portfolio of interventions, including intensified screening, rapid treatment initiation, partner services and doxy-PEP, individually and in combination. Using data-driven calibration and scenario analyses, we estimate the reproduction number, quantify the contribution of reinfection, evaluate the epidemiological impact of intervention strategies, and assess robustness to behavioral shifts that increase transmission risk. This work advances the existing literature by jointly representing behavioral, clinical, and biomedical drivers of syphilis transmission in a U.S. MSM context. By quantifying how reinfection, treatment performance, behavior and doxy-PEP interact, our findings aim to inform intervention prioritization and guide programmatic decision-making for MSM-focused syphilis control. The modeling framework provides a flexible platform for future extensions, including jurisdiction-specific analyses. The novelty of this study lies in the integration of reinfection dynamics, treatment heterogeneity, behavioral adaptation, and data-driven calibration within a unified syphilis transmission framework. While previous studies have examined individual aspects of syphilis transmission, such as reinfection dynamics, staged progression, or intervention strategies, our contribution lies in integrating these features within a single unified framework. Specifically, the proposed model simultaneously incorporates reinfection, two-stage treatment aligned with clinical practice, treatment failure, and behavioral adaptation within a data-driven setting calibrated to U.S. MSM surveillance data. This integrated approach enables the investigation of interaction effects among these mechanisms, particularly how reinfection and behavioral responses jointly influence persistence and threshold dynamics. As a result, the model provides new insights into conditions under which standard control thresholds may fail and highlights the importance of combined intervention strategies.

The remainder of the paper is organized as follows. Section 2 presents the model formulation. Section 3 provides analytic results. Section 4 details the joint intervention analyses, while Section 5 reports numerical simulations and scenario analyses. Section 6 concludes with key findings and implications for future research.

## 2 Mathematical model

### 2.1 Model description

The system of equations presented models the transmission dynamics of syphilis within a closed human population. The total population is divided into five epidemiological compartments at any time *t*: susceptible individuals (*S*(*t*)), early infectious individuals (*I*(*t*)), late infectious individuals (*L*), and treatment/recovery classes (*R*_1_(*t*)) and *R*_2_(*t*) are Recovered (at-risk) individuals under routine follow-up who can still be reinfected by *I*(*t*) or *L*(*t*). The total human population at any time *t* is thus given by *N*(*t*) = *S*(*t*)+*I*(*t*)+*L*(*t*)+*R*_1_(*t*)+*R*_2_(*t*).

The model accounts for natural birth and death processes, disease progression, treatment, temporary immunity, and reinfection. The susceptible population *S* increases through recruitment at a rate Λ and decreases due to infection through contact with early or late-stage infectious individuals. The force of infection is modeled as β_1_*S*(*I*+ξ*L*), where β_1_ is the effective contact rate and ξ captures the relative infectiousness of late-stage individuals compared to early-stage cases. Susceptible individuals may also arise from those who lose temporary immunity, transitioning from the *R*_2_ class at rate δ, and are removed through natural death at rate μ. Early infectious individuals (*I*) are generated through primary infection of susceptibles and reinfection of individuals in the recovery class *R* = *R*_1_+*R*_2_, modeled via β_2_*R*(*I*+ξ*L*). These individuals exit the compartment through treatment at a rate γ_*t*_ under control effort *u*_1_, or progress to late-stage infection if not successfully treated. The parameter *p* represents the proportion of individuals who are treated and return to temporary immunity, while (1−*p*) reflects those who fail treatment and progress to the late stage. Untreated individuals also progress at a rate γ_1_. All classes are subject to natural death at a rate μ. Late infectious individuals (*L*) accumulate from failed or missed treatment of early infectious individuals and are treated at a rate γ_2_ under control *u*_1_. Successfully treated late-stage individuals transition to *R*_1_. The recovery class *R*_1_ consists of individuals who have recovered from early or late infection. These individuals can be reinfected and move back to the *I* class or progress to the *R*_2_ class at rate *u*_2_, from which they may lose immunity at rate δ and become susceptible again. This model structure is supported by empirical findings indicating that individuals in the late latent stage can remain infectious, albeit at reduced levels (modeled through ξ) (34). Treatment parameters (γ_*t*_, γ_1_, γ_2_) are justified based on clinical recovery durations and treatment protocols reported in syphilis management guidelines (35). The reinfection of treated individuals (β_2_) is consistent with observed recurrent infections, especially in high-risk populations. We assume that the inequality γ_*t*_≥γ_1_ reflects that treatment is assumed to be at least as effective as the natural recovery or progression rate, ensuring that control interventions have a non-negative impact. The state variables and model parameters are given in Tables 1, 2.

**Table 1:** Description of state variables and their biological interpretations.

State variables	Biological meaning
*S*(*t*)	The susceptibles humans
*I*(*t*)	The primary and secondary infection stage
*L*(*t*)	The late-stage or untreate stage
*R*_1_(*t*)	The Recovered (at-risk) individuals
*R*_2_(*t*)	The Recovered (immune) with temporary immunity

**Table 2:** Model parameters and their biological interpretations in the syphilis transmission model with control.

Parameter	Description
Λ	Recruitment rate
γ_1_	Natural recovery rate
δ	Rate of waning immunity
μ	Natural mortality rate
β_1_	Effective transmission rate
β_2_	Effective reinfection rate
*a*	Behavioral or saturation parameter
γ_*t*_	Treatment rate for infectious individuals
γ_2_	Treatment rate for latent individuals
ξ	Relative infectiousness of latent individuals
*u*_1_	Treatment coverage for infectious and latent individuals
*u*_2_	Enhanced recovery or transition from *R*_1_ to *R*_2_
ψ	Modification parameter for the latent individuals
*p*	Probability of successful treatment of infectious individuals

Hence, the mathematical model is given by


$$\{\begin{array}{lll} \frac{dS}{dt} & = & \Lambda -\beta_{1}S(I+\xi L)+\delta R_{2}-\mu S,\\ \frac{dI}{dt} & = & \beta_{1}S(I+\xi L)+\beta_{2}R(I+\xi L)-pu_{1}\gamma_{t}I-(1-p)u_{1}\gamma_{t}I\\ \; & - & (1-u_{1})\gamma_{1}I-\mu I,\\ \frac{dL}{dt} & = & (1-p)u_{1}\gamma_{t}I+(1-u_{1})\gamma_{1}I-u_{1}\gamma_{2}L-\mu L,\\ \frac{dR_{1}}{dt} & = & pu_{1}\gamma_{t}I+u_{1}\gamma_{2}L-\beta_{2}R(I+\xi L)-u_{2}R_{1}-\mu R_{1},\\ \frac{dR_{2}}{dt} & = & u_{2}R_{1}-\delta R_{2}-\mu R_{2}. \end{array}$$ (1)

with an assumption that γ_*t*_≥γ_1_ and the initial conditions*S*(0)>0, *I*(0)≥0, *L*(0)≥0, *L*(0)≥0, *R*_1_(0)≥0, *R*_2_(0)>0.

### 2.2 Incorporating behavior change function

To incorporate behavioral adaptation, we assume that the effective risky contact rate decreases as the perceived prevalence of infection increases. In the present STI setting, this reduction may reflect fewer sexual contacts, fewer partners, greater caution in partner selection, or other risk-avoidance responses triggered by awareness of ongoing transmission. Therefore, instead of using the bilinear infection term alone, we scale the transmission pressure by a decreasing prevalence-dependent response function:


$$\begin{array}{l} f\left(I,L\right)=\frac{1}{1+a\left(I+\psi L\right)} \end{array}$$ (2)

where *a*>0 is a sensitivity parameter representing how strongly individuals reduce contact with increasing prevalence and ψ is a weighting factor describing the contribution of the latent class *L* to perceived risk. Hence, the modified force of infection becomes


$$\begin{array}{l} \frac{\beta_{1}S\left(I+\xi L\right)}{1+a\left(I+\psi L\right)}, \end{array}$$

with ξ, as explained before, measures the contribution of *L* to transmission. Therefore, in our model, ξ and ψ represent two different things, where ξ describes the contribution of *L* in the transmission, whereas ψ measures its contribution to behavioral awareness. Biologically, this distinction is reasonable because latent individuals may contribute less to infectiousness than symptomatic or fully infectious individuals, while still contributing to perceived disease burden through diagnosis, contact tracing, or public awareness. The function *f*(*I, L*) is positive, bounded, and monotonically decreasing with respect to (*I*+ψ*L*). Thus, as disease prevalence increases, the effective transmission rate is reduced but never becomes negative. This type of rational decreasing function is commonly used in epidemiological modeling to represent saturation effects, psychological responses, and adaptive reductions in effective contact rates as the disease burden grows. In this way, the model captures prevalence-driven behavioral feedback while preserving mathematical tractability.

**Figure A1:**
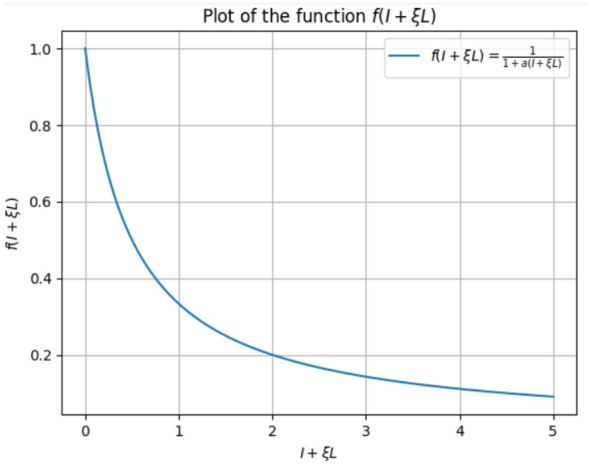
Behavior function model diagram.

The model behavior change function is depicted in Figure A1 The system of equations the model dynamics, as explained above while substituting (2) and update (1), thus given below as a non-linear ODE;


$$\{\begin{array}{lll} \frac{dS}{dt} & = & \Lambda -\frac{\beta_{1}S(I+\xi L)}{1+a(I+\psi L)}+\delta R_{2}-\mu S,\\ \frac{dI}{dt} & = & \frac{\beta_{1}S(I+\xi L)}{1+a(I+\psi L)}+\frac{\beta_{2}R(I+\xi L)}{1+a(I+\psi L)}\\ \; & - & pu_{1}\gamma_{t}I-(1-p)u_{1}\gamma_{t}I-(1-u_{1})\gamma_{1}I-\mu I,\\ \frac{dL}{dt} & = & (1-p)u_{1}\gamma_{t}I+(1-u_{1})\gamma_{1}I-u_{1}\gamma_{2}L-\mu L,\\ \frac{dR_{1}}{dt} & = & pu_{1}\gamma_{t}I+u_{1}\gamma_{2}L-\frac{\beta_{2}R_{1}(I+\xi L)}{1+a(I+\psi L)}-u_{2}R_{1}-\mu R_{1},\\ \frac{dR_{2}}{dt} & = & u_{2}R_{1}-\delta R_{2}-\mu R_{2}. \end{array}$$ (3)

subject to the initial conditions


$$\begin{array}{l} S\left(0\right)>0,I\left(0\right)\geq 0,L\left(0\right)\geq 0,L\left(0\right)\geq 0,R_{1}\left(0\right)\geq 0,R_{2}\left(0\right)>0. \end{array}$$

## 3 Model analysis

Consider the system in Equation (3). We prove the existence and uniqueness of the solution in the next subsection below.

### 3.1 Positivity and boundedness of solutions

We define the total population as


$$\begin{array}{l} N\left(t\right)=S\left(t\right)+I\left(t\right)+L\left(t\right)+R_{1}\left(t\right)+R_{2}\left(t\right). \end{array}$$

Adding the five model equations yields


$$\begin{array}{l} \frac{dN}{dt}=\Lambda -\mu N. \end{array}$$

This is a linear ODE with a solution


$$\begin{array}{l} N\left(t\right)=\frac{\Lambda }{\mu }+\left(N\left(0\right)-\frac{\Lambda }{\mu }\right)e^{-\mu t}. \end{array}$$

As *t* → ∞, $N\left(t\right)\to \frac{\Lambda }{\mu }$. Hence, the total population is bounded.

Moreover, given non-negative initial conditions, all model variables remain non-negative for all *t*>0 by standard ODE theory. We define the positively invariant region


$$\begin{array}{l} \Omega =\left\{\left(S,I,L,R_{1},R_{2}\right)\in {\mathbb{R}}_{+}^{5}:N\left(t\right)\leq \frac{\Lambda }{\mu }\right\}. \end{array}$$

Thus, solutions starting in Ω remain in Ω for all future time.

### 3.2 The disease-free equilibrium

The first equilibrium point from our proposed model in system (Equation 3) is the disease-free equilibrium, denoted by $E_{0}$. This equilibrium represents a condition when no infected individuals exist in the population. This equilibrium is given by


$$\begin{array}{l} E_{0}=\left(S,I,L,R_{1},R_{2}\right)=\left(\frac{\Lambda }{\mu },0,0,0,0\right). \end{array}$$

It can be seen that all non-susceptible individuals are extinct from the population as *t* → ∞, and it leaves the Susceptible *S* compartment. Total population *N* in $E_{0}$ is given by $\frac{\Lambda }{\mu }$, which is the ratio between total recruitment rate and natural death rate.

### 3.3 The reproduction number

Before we proceed to the calculation of the non-trivial equilibrium, it is important to calculate the basic reproduction number of our model, which is denoted by $R_{0}$. The basic reproduction number represents the expected number of secondary cases in a virgin population that result from infection by one primary infected individual during its infectious period. Since our model involves control variables *u*_1_ and *u*_2_, which are non-negative, we will calculate the control reproduction number of our model, rather than the basic reproduction number, although the calculation methods are the same. The basic reproduction number of our model can be calculated by taking *u*_1_ = *u*_2_ = 0 and substituting it into the control reproduction number.

We use the next-generation matrix approach, introduced by Diekmann et al. (36). Please see (37–39) for more examples of the implementation of this method in other epidemic modeling. To calculate the next-generation matrix, we calculate the transition matrix from the infected compartments of model Equation (3), and yield


$$\begin{array}{l} V=\left[\begin{array}{cc} -u_{1}\gamma_{t}-\left(1-u_{1}\right)\gamma_{1}-\mu & 0\\ \left(1-p\right)u_{1}\gamma_{t}+\left(1-u_{1}\right)\gamma_{1} & -u_{1}\gamma_{2}-\mu \end{array}\right], \end{array}$$

while the transmission matrix is:


$$\begin{array}{l} F=\left[\begin{array}{cc} \frac{\beta_{1}\Lambda }{\mu } & \frac{\beta_{1}\Lambda \xi }{\mu }\\ 0 & 0 \end{array}\right]. \end{array}$$

Hence, with *E* = [10]^*T*^, we have the next-generation matrix with small domain given by


$$\begin{array}{l} NGM=-E^{T}{\mathrm{FV}}^{-1}E=\left[\frac{\Lambda \beta_{1}\left(\left(1-u_{1}\right)\xi \gamma_{1}++\left(1-p\right)u_{1}\xi \gamma_{t}+u_{1}\gamma_{2}+\mu \right)}{\mu \left(u_{1}\gamma_{2}+\mu \right)\left(\left(1-u_{1}\right)\gamma_{1}+u_{1}\gamma_{t}+\mu \right)}\right]. \end{array}$$

Hence, the controlled reproduction number is given by:


$$R_{c}=\frac{\Lambda \beta_{1}\left(\left(1-u_{1}\right)\xi \gamma_{1}++\left(1-p\right)u_{1}\xi \gamma_{t}+u_{1}\gamma_{2}+\mu \right)}{\mu \left(u_{1}\gamma_{2}+\mu \right)\left(\left(1-u_{1}\right)\gamma_{1}+u_{1}\gamma_{t}+\mu \right)}.$$

It is important to notice that we have no *u*_2_ appearing in the expression of $R_{0}$. Furthermore, if we substitute *u*_1_ = 0, then the controlled reproduction number reduces to the basic reproduction number, which is given by:


$$R_{0}=\frac{\Lambda \beta_{1}\left(\xi \gamma_{1}+\mu \right)}{\mu^{2}\left(\mu +\gamma_{1}\right)}.$$

We can therefore interpreter the epidemiological meaning of $R_{0}$ as follows; Given that


$$\begin{array}{l} R_{0}=\underset{\text{Infections from the infectious stage}\left(I\right)}{\underset{︸}{\frac{\beta_{1}\Lambda }{\mu \left(\mu +\gamma_{1}\right)}}}+\underset{\text{Additional Infections via the latent stage}\left(L\right)}{\underset{︸}{\frac{\beta_{1}\Lambda }{\mu \left(\mu +\gamma_{1}\right)}\cdot \frac{\xi \gamma_{1}}{\mu }}}. \end{array}$$

The first term counts infections generated during the average infectious duration $\frac{1}{\left(\mu +\gamma_{1}\right)}$. The second adds infections from those who progress to *L* (fraction $\frac{\gamma_{1}}{\left(\mu +\gamma_{1}\right)}$), spend on average $\frac{1}{\mu }$ time there, and transmit at relative infectiousness ξ. Hence, $R_{0}<1$ implies fade-out, whereas $R_{0}>1$ implies persistence.

### 3.4 Endemic equilibrium

The endemic equilibrium is given as follows


$$\begin{array}{l} E_{+}=\left(S,I,L,R_{1},R_{2}\right)=\left(S_{+},L_{+},I_{+},R_{1_{+}},R_{2_{+}}\right) \end{array}$$

with


$$\begin{array}{lll} S_{+} & = & \frac{\left(\Lambda +\delta R_{2_{+}}\right)\left(1+aI_{+}+\psi aL_{+}\right)}{\mu +\beta_{1}I_{+}+\beta_{1}\xi L_{+}+\mu aI_{+}+\psi \mu aL_{+}},\\ L_{+} & = & \frac{I_{+}\left(\gamma_{1}+\gamma_{t}p\right)\left(1-u_{1}\right)}{\mu +\gamma_{2}u_{1}}, \end{array}$$

$$\begin{array}{l} R_{1_{+}}=\frac{u_{1}\left(I_{+}p\gamma_{t}+L_{+}\gamma_{2}\right)\left(L_{+}a\psi +I_{+}a+1\right)}{L_{+}a\mu \psi +L_{+}a\psi u_{2}+I_{+}a\mu +I_{+}au_{2}+L_{+}\xi \beta_{2}+I_{+}\beta_{2}+\mu +u_{2}}, \end{array}$$

$$\begin{array}{l} R_{2_{+}}=\frac{u_{2}R_{1_{+}}}{\mu +\delta }, \end{array}$$

where *I*_+_ is a positive solution from the following two-degree polynomial


$$f\left(I\right)=a_{2}I^{2}+a_{1}I+a_{0}=0,$$

with $a_{0}=\mu \left(\mu +u_{2}\right)\left(\mu +\delta \right){\left(\mu +u_{1}\gamma_{2}\right)}^{3}\left(\gamma_{1}\left(1-u_{1}\right)+\mu +u_{1}\gamma_{t}\right)\left(1-R_{0}\right).$ Note that *a*_2_ and *a*_1_ have a long expression to be shown in this article. However, it can be shown numerically that *a*_2_ is always positive. Therefore, we will always have a unique endemic equilibrium whenever $R_{0}>1$. Furthermore, since *f*(*I*) is a two-degree polynomial, it is possible to have two positive roots of *f*(*I*). To analyze this further, we use a similar approach as in Aldila et al. (40), we analyze the possible sign of $\frac{\partial I}{\partial R_{0}}$ at $R_{0}=1,I=0$. If the sign is positive, then we will have no endemic equilibrium for any value $R_{0}<1$. On the other hand, if the sign is negative, then we will always have two endemic equilibria for $R_{0}\in \left[FP,1\right]$ where FP is the fold point that satisfies the discriminant of *F*(*I*) = 0. Further experiments will be shown numerically in the proceeding section.

### 3.5 Stability of $E_{0}$

Linearization of system Equation (3) in $E_{0}$ is given by:


$$\begin{array}{l} J\left(E_{0}\right)=\left[\begin{array}{ccccc} -\mu & -\frac{\beta_{1}\Lambda \xi }{\mu } & -\frac{\beta_{1}\Lambda }{\mu } & 0 & \delta \\ 0 & -\gamma_{2}u_{1}-\mu & \left(1-p\right)u_{1}\gamma_{t}+\left(1-u_{1}\right)\gamma_{1} & 0 & 0\\ 0 & \frac{\beta_{1}\Lambda \xi }{\mu } & \frac{\beta_{1}\Lambda }{\mu }-u_{1}\gamma_{t}-\left(1-u_{1}\right)\gamma_{1}-\mu & 0 & 0\\ 0 & \gamma_{2}u_{1} & pu_{1}\gamma_{t} & -u_{2}-\mu & 0\\ 0 & 0 & 0 & u_{2} & -\delta -\mu \end{array}\right]. \end{array}$$

It can be seen from the matrix that we have three explicit eigenvalues, i.e., −μ, −(δ+μ), and −(*u*_2_+μ), all of which are negative. The other two eigenvalues are coming from the following polynomial:


$$\begin{array}{l} g\left(\lambda \right)=\mu \lambda^{2}+\left(\gamma_{1}\mu \left(1-u_{1}\right)+2\mu^{2}+\mu u_{1}\left(\gamma_{t}+\gamma_{2}\right)-\beta_{1}\Lambda \right)\lambda \\ \;+\left(\gamma_{2}u_{1}+\mu \right)\left(\gamma_{1}\left(1-u_{1}\right)+\mu +u_{1}\gamma_{t}\right)\left(1-R_{0}\right)\mu =0. \end{array}$$

It can be seen that each coefficient of *g*(λ) will be positive if and only if $R_{0}<1$. Hence, we have the following theorem.

**Theorem 1**. *The disease-free equilibrium $E_{0}$ of system Equation (3) is locally asymptotically stable if* $R_{0}<1$, *and unstable if* $R_{0}>1$.

### 3.6 Stability of $E_{1}$ and bifurcation analysis numerically

We use the following parameter values to analyze the stability and construct the bifurcation diagram of system Equation (3), with β_1_ serving as the bifurcation parameter:


$$\begin{array}{l} \Lambda =10,000/76.4,\mu =1/76.4,\beta_{2}=0.1,u_{1}=0.2,u_{2}=0.2,\\ \;p=0.5,\gamma_{1}=3.65,\gamma_{2}=4,\gamma_{t}=8,\delta =0.5,\xi =0.6, \end{array}$$

ψ = 0.6.

**Figure 1:**
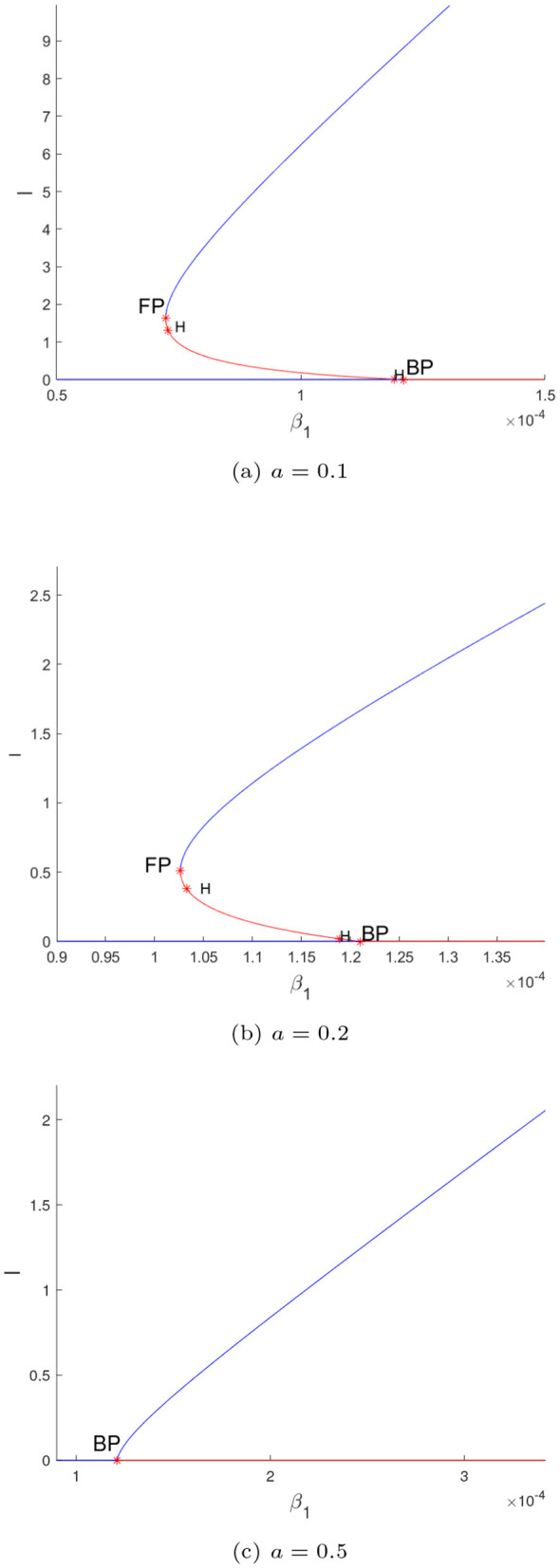
Backward bifurcation diagram of system Equation (3) with β_1_ as the bifurcation parameter and with various values of *a*. Red and blue curve represent the unstable and stable branch of equilibrium curve. FP, BP, and H represent the fold point, branching point, and hopf point, respectively. **(a)** *a* = 0.1. **(b)** *a* = 0.2. **(c)** *a* = 0.5.

The result of the continuation process using *MatCont* is shown in Figure 1.

With the above parameter values, we find that the branching point (BP), corresponding to $R_{0}=1$, occurs at β_1_ = 0.000121. The expression $R_{0}\left(\beta_{1}\right)=8261.67\beta_{1}$ remains unchanged for any chosen value of *a* because *a* does not appear in the formula for $R_{0}$. The second important point is the Fold Point (FP), obtained from the discriminant of *f*(λ, β_1_) = 0. Substituting the above parameter values into *f*(λ) = 0, we obtain the discriminant:


$$\begin{array}{l} D\left(\beta_{1},a\right)=\left(11641.1a^{2}+10947.6a+2556.8\right)\beta_{1}^{2}\\ \;+\left(10^{-8}a^{2}-2.62a-0.01\right)\beta_{1}+9.73\times 10^{-9}\\ \;-2\times 10^{-15}a-1.6\times 10^{-12}a^{2}. \end{array}$$

By substituting *a* = 0.1 and *a* = 0.2 into *D*(β_1_, *a*) = 0 and solving for β_1_, we find that the Fold Points occur at β_1_ = 0.0000723 and β_1_ = 0.000102, respectively. For *a* = 0.5, β_1_ corresponding to the Fold Point does not yield a positive solution for *f*(*I*) = 0, and thus does not satisfy the positivity criterion.

Figure 1 illustrates how the equilibrium *I* of system Equation (3) behaves with respect to variations in β_1_, for example, when *a* = 0.5. We observe that for any β_1_ < BP, $R_{0}<1$, and the disease-free equilibrium $E_{0}$ remains stable. This stability changes as β_1_ crosses BP at β_1_ = 0.000121. Beyond this point, the disease-free equilibrium becomes unstable, and an endemic equilibrium emerges. The value of *I* in the endemic equilibrium increases as β_1_ increases, and this endemic equilibrium remains stable for all β_1_ values greater than BP.

A different behavior appears for smaller values of *a*, such as *a* = 0.1 or *a* = 0.2. For *a* = 0.1, the disease-free equilibrium remains stable when β_1_ < BP. However, unlike the case of *a* = 0.5, an additional stable equilibrium can exist for β_1_ < BP, specifically for β_1_∈[FP, BP]. As β_1_ increases from zero, only the stable disease-free equilibrium exists. As β_1_ approaches FP, a hysteresis phenomenon occurs, marked by a sudden transition from zero to a positive equilibrium value. From FP onward, three equilibria coexist: one stable endemic equilibrium, one unstable endemic equilibrium, and one stable disease-free equilibrium. The stable endemic equilibrium increases in magnitude with β_1_, while the unstable endemic equilibrium decreases and disappears at BP. At BP, the disease-free equilibrium loses stability and the unstable endemic equilibrium vanishes, resulting in a bistability phenomenon for β_1_∈[FP, BP].

This backward bifurcation indicates that when $R_{0}<1$, disease eradication is not always guaranteed. A similar explanation applies for the case *a* = 0.2.

To enhance our understanding of the dynamical properties of the proposed model, we extend our analysis by constructing a codimension-2 bifurcation diagram, using β_1_ and *u*_1_ as the bifurcation parameters, while varying *u*_2_ and *a* to identify the type of bifurcation that may occur at $R_{0}=1$. First, we employ the same parameter values as those used to produce Figure 1a, except for *u*_2_ and *a*, which are treated as free variables:


$$\begin{array}{l} \Lambda =10000/76.4,\mu =1/76.4,\beta_{2}=0.1,p=0.5,\gamma_{1}=3.65,\\ \;\gamma_{2}=4,\gamma_{t}=8,\delta =0.5, \end{array}$$

ξ = 0.6, ψ = 0.6. Substituting these parameter values into the expression of $R_{0}$, we obtain:


$$\frac{10000.0\beta_{1}\left(4.210000000u_{1}+2.203089005\right)}{\left(\frac{87u_{1}}{20}+3.663089005\right)\left(4u_{1}+0.01308900524\right)}=1.$$

By setting *u*_1_ = 0.2, we find that β_1_ = 0.00007234546342 yields $R_{0}=1$. Substituting this parameter value into $\frac{\partial I}{\partial R_{0}}$, we have


$$\begin{array}{l} \frac{\partial I}{\partial R_{0}}<0\Leftrightarrow 0.06128719776au_{2}+0.0008021884534a\\ \;+0.00002334884897u_{2}-0.006022642151<0. \end{array}$$

**Figure 2:**
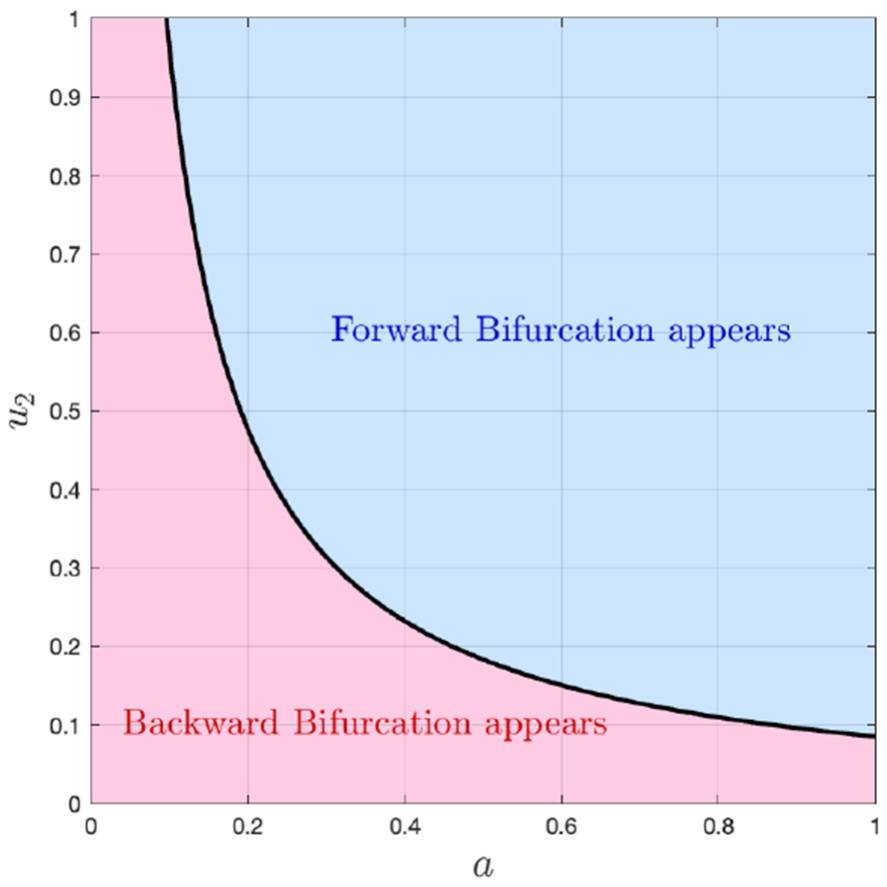
Regions indicating the type of bifurcation in system Equation (3). The black curve represents the locus where$\frac{\partial I}{\partial R_{0}}\left(R_{0}=1,I=0\right)=0.$ The red and blue areas correspond to combinations of *a* and *u*_2_ that result in backward and forward bifurcations, respectively.

Plotting the above inequality in the (*a, u*_2_)-plane provides the regions corresponding to backward or forward bifurcation at $R_{0}=1$, as shown in Figure 2. It is evident that smaller values of *a* and *u*_2_ increase the likelihood of a backward bifurcation, which induces the existence of an endemic equilibrium even when $R_{0}$ is already less than one.

**Figure 3:**
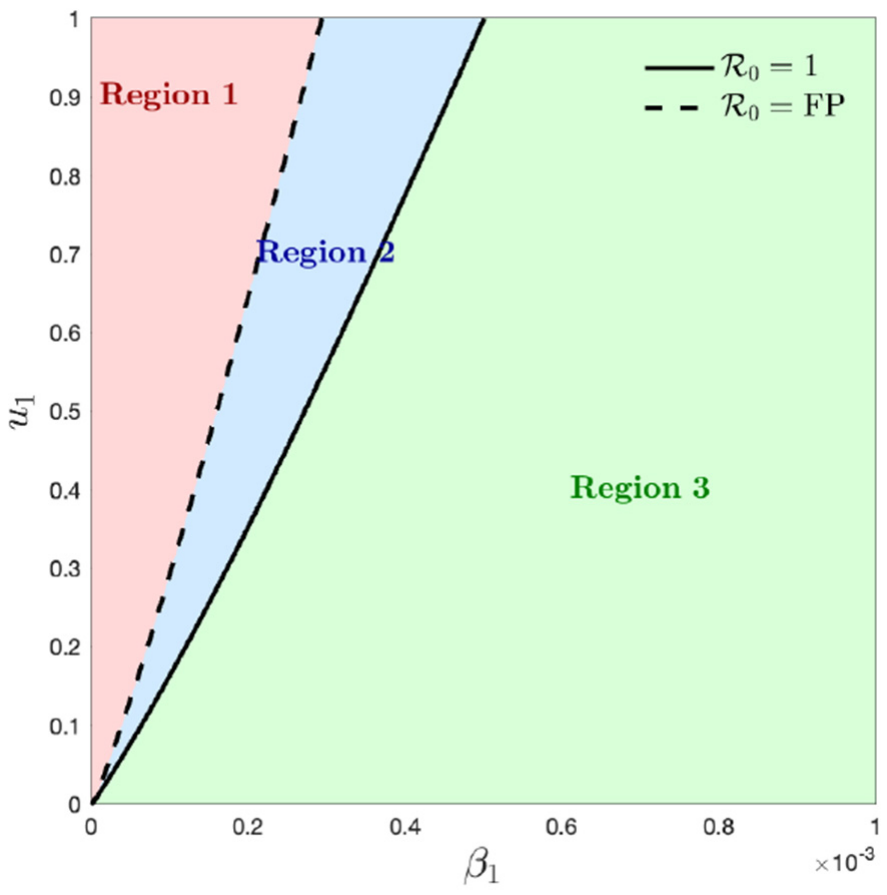
Codimension-2 bifurcation diagram with β_1_ and *u*_1_ as the bifurcation parameters.

**Figure 4:**
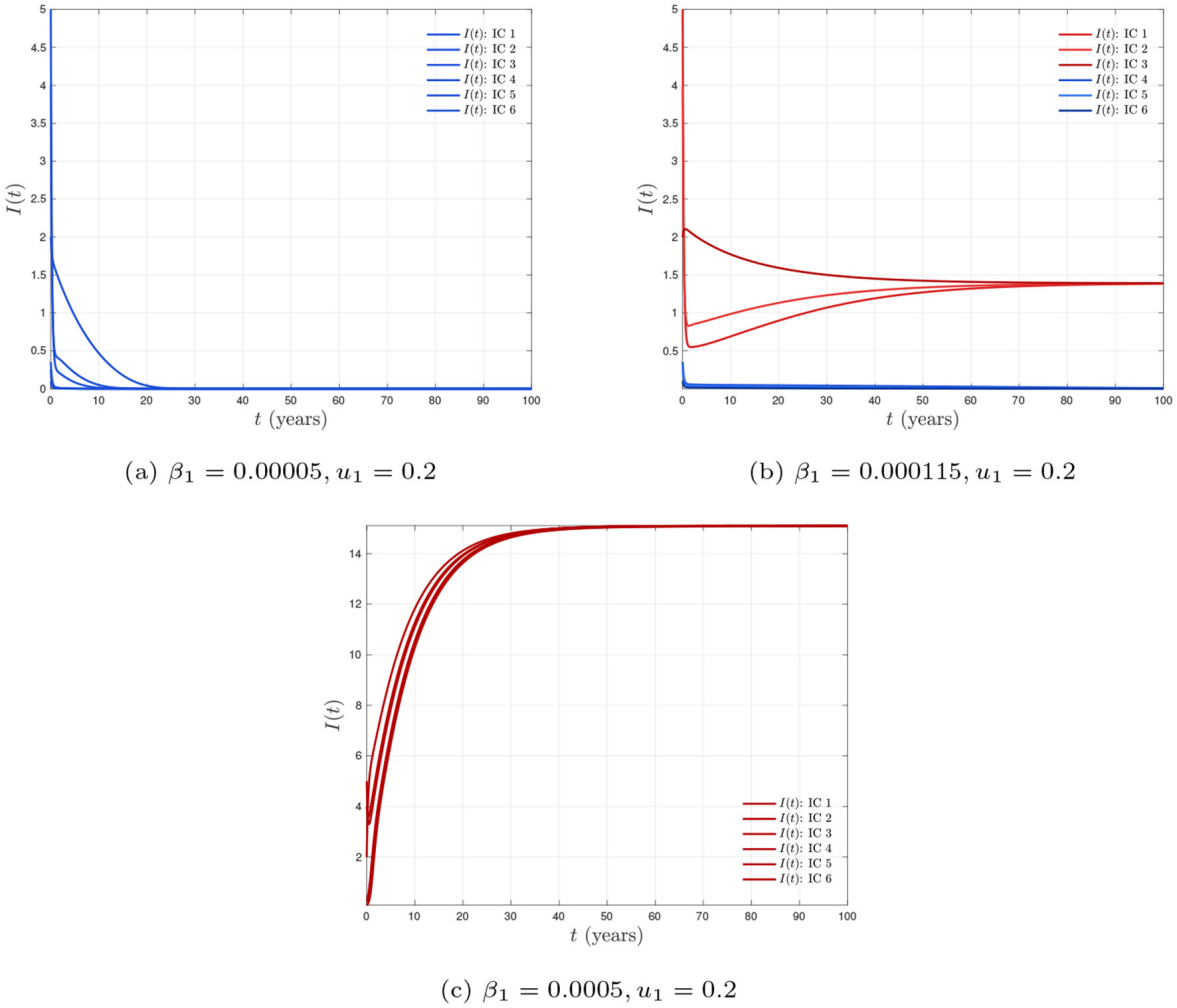
Autonomous simulation of system Equation (3) when the parameter combination gives a stability only in disease-free equilibrium in **(a)**, bistability in **(b)**, and endemic equilibrium point in **(c)**. Red and blue curves present a solution that tends to the endemic equilibrium or the disease-free equilibrium, respectively.

With the results illustrated in Figure 2, suitable combinations of *a* and *u*_2_ can be selected to induce either a backward or a forward bifurcation. Specifically, when *a* = 0.2 and *u*_2_ = 0.2, the system exhibits a backward bifurcation, as depicted in Figure 1b, where β_1_ serves as the bifurcation parameter. Furthermore, by considering β_1_ and *u*_1_ as the bifurcation parameters, the corresponding regions of existence and stability of equilibria for this parameter configuration are presented in Figure 3. The diagram reveals three distinct regions, each associated with a different number and type of equilibrium points. In Region 1 (red area), where $R_{0}<FP$, the model admits no endemic equilibrium, and the disease-free equilibrium remains asymptotically stable. In Region 2 (blue area), where $R_{0}\in \left[FP,1\right]$, the system displays bistability: a stable disease-free equilibrium coexists with both a stable and an unstable endemic equilibrium. Finally, in Region 3 (green area), where $R_{0}>1$, the disease-free equilibrium loses stability, and a single stable endemic equilibrium persists. To further illustrate the qualitative behavior of the system, three autonomous simulation scenarios—each corresponding to a representative sampling point from the three identified regions—are presented in Figure 4.

These simulations demonstrate how the system dynamics depend on both the parameter values and the initial conditions. In panels (a) and (c), all trajectories originating from different initial conditions converge to the same stable equilibrium, corresponding respectively to the disease-free and endemic equilibrium. In contrast, the trajectory shown in panel (b) exhibits strong dependence on the initial condition, reflecting the occurrence of a bistability phenomenon. This bistability implies that the long-term outcome of the system can vary between disease eradication and persistence, depending on the initial distribution of the population among compartments, even under identical parameter settings. Biologically, these results highlight the critical role of parameters *a* and *u*_2_ in determining the transition dynamics around the threshold $R_{0}=1$. The presence of a backward bifurcation implies that disease persistence can occur even when $R_{0}<1$, emphasizing that simply reducing the basic reproduction number below one may not be sufficient for disease eradication.

The occurrence of backward bifurcation has important epidemiological implications. In classical epidemic models, the condition $R_{0}<1$ is sufficient to ensure disease elimination. However, in the presence of backward bifurcation, this condition is no longer sufficient, as a stable endemic equilibrium may coexist with the disease-free equilibrium when $R_{0}<1$. In the context of sexually transmitted infections, this means that reducing transmission through partial interventions such as moderate condom use, limited behavioral change, or incomplete screening coverage may not be adequate to eliminate the infection. If the initial prevalence remains sufficiently high, the disease can persist even when $R_{0}$ is below unity. Hence, the interaction between awareness-related parameters (*a*, *u*_2_) and the transmission rate β_1_ plays a crucial role in shaping the system's long-term behavior and guiding effective control strategies. This finding highlights the importance of early intervention and sustained control efforts in high-risk populations.

In the next section 4, we carry out a joinpoint regression analysis using the MSM real dataset.

## 4 Data analysis

### 4.1 Joinpoint regression analysis

Joinpoint regression is a statistical approach used to identify and model significant changes in temporal trends (41, 42). It fits a continuous, piecewise log-linear function of time in which the points of slope change, called *joinpoints* or *changepoints*, are estimated from the data. Traditional linear or log-linear models assume a single, constant growth rate across the entire study period. However, epidemiological processes often exhibit phases of acceleration, stabilization, or decline due to changing behavior, prevention efforts, or reporting practices.

Joinpoint regression is used to objectively identify time periods in which significant trend changes occur and to estimate the corresponding Annual Percent Change (APC) for each segment. This approach is particularly valuable in infectious disease epidemiology because it enables:

(i) Detection of inflection points, that is, years in which the epidemic trajectory changes significantly.
(ii) Quantification of growth or decline through APC estimates for each trend segment.
(iii) Alignment with interventions, allowing interpretation of whether policy changes, treatment rollouts, or surveillance disruptions corresponded with observed shifts.
(iv) Policy relevance by providing clear, interpretable summaries (for example, APC increased by 15% per year from 2011 to 2016, but stabilized thereafter) that inform future planning.

Let *y*_*t*_ denote the observed number of cases in year *t*, and let *g*(·) represent the logarithmic link function. With *K* joinpoints occurring at times τ_1_ < ⋯ < τ_*K*_, the model is defined as


$$\begin{array}{l} \underset{g\left(\mu_{t}\right)}{\underset{︸}{\log\left\{E\left(y_{t}\right)\right\}}}=\beta_{0}+\beta_{1}t+\overset{K}{\underset{k=1}{\sum }}\gamma_{k}{\left(t-\tau_{k}\right)}_{+},\;{\left(x\right)}_{+}=\max\left(0,x\right),\; \end{array}$$ (4)

where each τ_*k*_ denotes a time at which the slope of the log-linear trend changes. This formulation represents a linear spline on the log scale, continuous in *t*, with distinct slopes for each segment. The segment-specific slopes on the log scale are


$$\begin{array}{l} \theta_{0}=\beta_{1},\;\theta_{j}=\beta_{1}+\overset{j}{\underset{k=1}{\sum }}\gamma_{k},\;j=1,\ldots ,K. \end{array}$$

For a segment with slope θ, the APC is defined as


$$\begin{array}{l} \text{APC}=(e^{\theta }-1)\times 100\%. \end{array}$$ (5)

An overall average APC over an interval [*t*_*a*_, *t*_*b*_] can be computed as a weighted average of the segment-specific slopes (weighted by segment length) and then transformed using Equation (5) (43). Uncertainty for both APC and AAPC is obtained via the delta method, using the covariance matrix of the estimated regression coefficients (43, 44).

### 4.2 Estimation, model selection, and results

Model Equation (4) is estimated using ordinary least squares on log(*y*_*t*_), which corresponds to assuming normally distributed errors on the log scale. All models with up to three joinpoints are evaluated, subject to a minimum of two observations per segment. The optimal model is selected by minimizing the Bayesian Information Criterion (BIC), which balances model fit and complexity. Confidence intervals for the APC are based on the normal approximation for the estimated slopes and the delta-method transformation in Equation (5). This BIC-based approach provides a computationally efficient and small-sample alternative to the permutation test procedure implemented in the U.S. National Cancer Institute (NCI) Joinpoint Regression Program (44, 45). We fit Equation (4) on log(cases) vs. calendar year using exhaustive search over *K* = 0, 1, 2, 3 joinpoints, BIC model selection, and delta-method CIs for APC. Minimum segment length was two observations. The selected joinpoints were ${\hat{\tau }}_{1}=2017$, ${\hat{\tau }}_{2}=2019$, and ${\hat{\tau }}_{3}=2021$. We analyzed USA MSM syphilis totals from 2011 to 2023. The BIC-selected model included three joinpoints at 2017, 2019, and 2021, producing four log–linear segments

**Figure 5:**
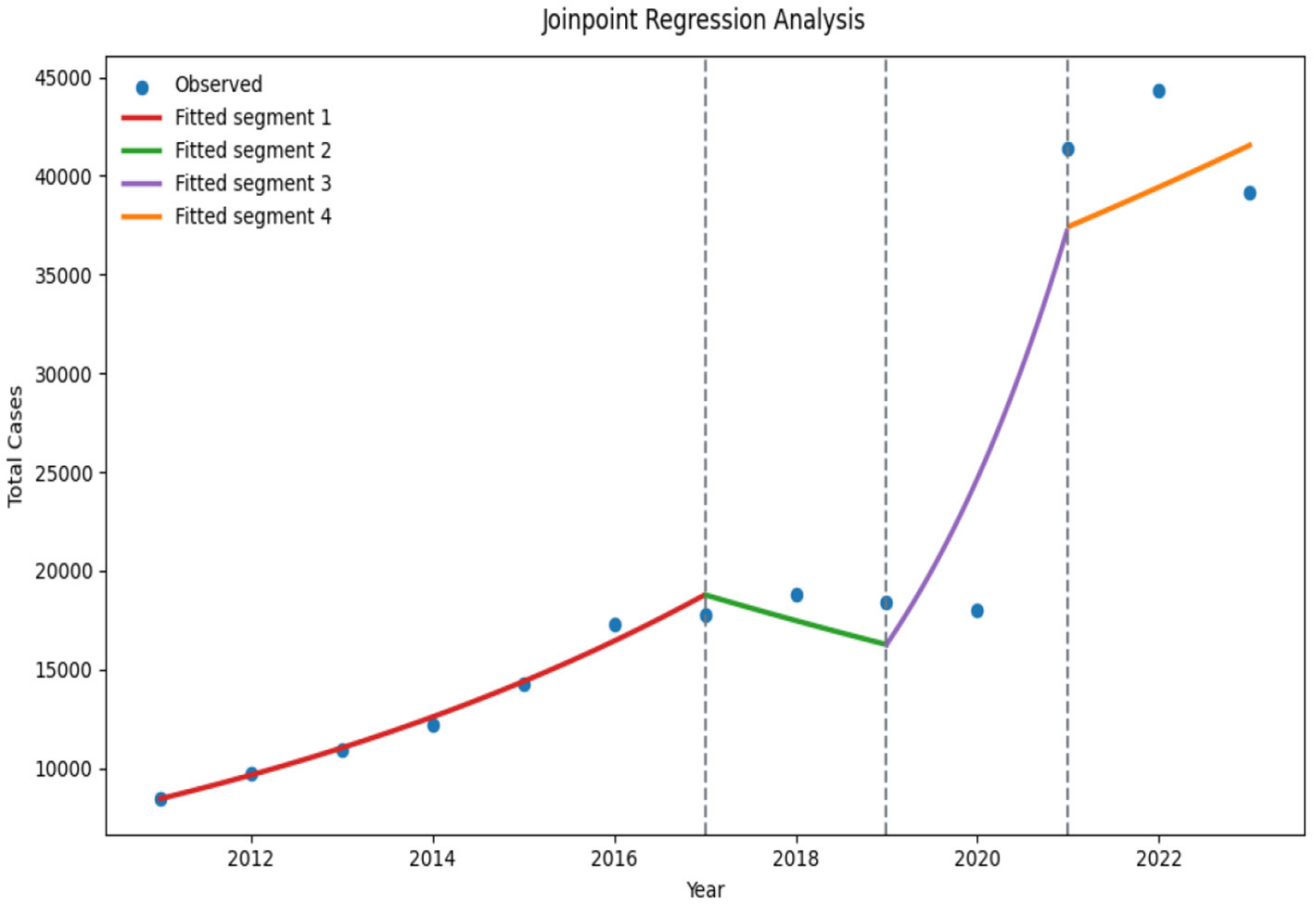
Jointpoint regression analysis showing different segments for the MSM yearly incidence cases from 2011 to 2023.

In Figure 5, between 2011 and 2017, syphilis cases among MSM in the USA increased at an average APC of +14.2%. This reflects a period of rapid and sustained growth, consistent with rising incidence during these years. From 2017 to 2019, the trend shifted, showing an APC of -7.0%. While this suggests a plateau or possible decline, the wide confidence interval overlapping zero indicates that the change was not statistically conclusive. The period 2019 to 2021 marked a dramatic inflection, with an APC of +51.7%. This represents an exceptional surge in cases, signaling a sharp and significant increase. In addition, from 2021 to 2023, the APC moderated to +5.4%. With confidence intervals that include zero, this phase points to a modest stabilization, consistent with the epidemic settling after the sharp rise. Moreover, the epidemic shows three distinct inflection points: a strong pre-2017 expansion, a 2017–2019 plateau/decline, an exceptional 2019–2021 upswing, and a post-2021 stabilization. These shifts are epidemiologically plausible given changes in testing, reporting disruptions, behavioral dynamics, and public health responses in this period. The joinpoint locations provide objective break dates that can be used to align policy timelines, model calibration windows, or intervention analyses. Joinpoint regression allows us to move beyond a single summary growth rate and to instead characterize the dynamic, piecewise nature of syphilis transmission among MSM in the USA.

Table 3 shows four distinct phases in syphilis trends among MSM from 2011 to 2023. Syphilis incidence rose significantly from 2011 to 2017, with an APC of 14.24% and a confidence interval that excludes zero, indicating a sustained increase. From 2017 to 2019, the APC was 6.95%, but the wide confidence interval crossing zero suggests no statistically meaningful decline. A marked resurgence occurred between 2019 and 2021, where the APC reached 51.71% with a strongly positive confidence interval, indicating a rapid and significant rise. In contrast, the period from 2021 to 2023 showed a modest APC of 5.37%, but the confidence interval again included zero, suggesting relative stabilization. These findings reflect an initial period of steady growth, an uncertain slowdown, a sharp epidemic surge, and subsequent stabilization in recent years. An important advantage of joinpoint regression is its ability to detect data-driven changes in epidemic trends over time. Although it is not directly incorporated into the mechanistic model, it serves as a complementary tool that provides valuable context for model interpretation and calibration. In our analysis (Figure 5 and Table 3), the estimated breakpoints (2017, 2019, and 2021) define four distinct phases of the epidemic: sustained growth from 2011–2017 (APC = 14.24%), an inconclusive decline or plateau from 2017–2019 (APC = −6.95%), a sharp surge from 2019–2021 (APC = 51.71%), and relative stabilization from 2021–2023 (APC = 5.37%). These phases provide objective, data-driven time intervals that inform model calibration and interpretation. In particular, they guide parameter estimation across periods, support scenario and sensitivity analyses, and help assess how transmission intensity varies over time. The identified breakpoints are also consistent with changes in behavior, testing practices, public health interventions, and reporting dynamics. By aligning the mechanistic model with these empirically identified phases, we enhance the interpretability of model results and situate the analysis within the observed, time-varying dynamics of syphilis transmission among MSM in the USA.

**Table 3:** Estimated annual percent change by joinpoint segment, 2011–2023.

Years	Log-slope $\hat{\theta }$	APC (%)	95% lower CI	95% upper CI
2011–2017	0.1332	14.24	8.68	20.09
2017–2019	−0.0720	−6.95	−20.19	8.48
2019–2021	0.4168	51.71	27.34	80.73
2021–2023	0.0523	5.37	−12.33	26.66

To carry out numerical simulations and parameter estimation using the epidemiological model Equation (3), we smoothed the yearly data, which were unevenly sparse, and used the cumulative cases to calibrate the model. The identified joinpoints (2017, 2019, and 2021) are used to inform calibration phases and interpret temporal changes in transmission dynamics within the compartmental model fitted in the next Section 5. The remaining simulations were then performed as presented in the next Section 5.

## 5 Numerical simulation

### 5.1 Model parameterization and fitting

**Figure 6:**
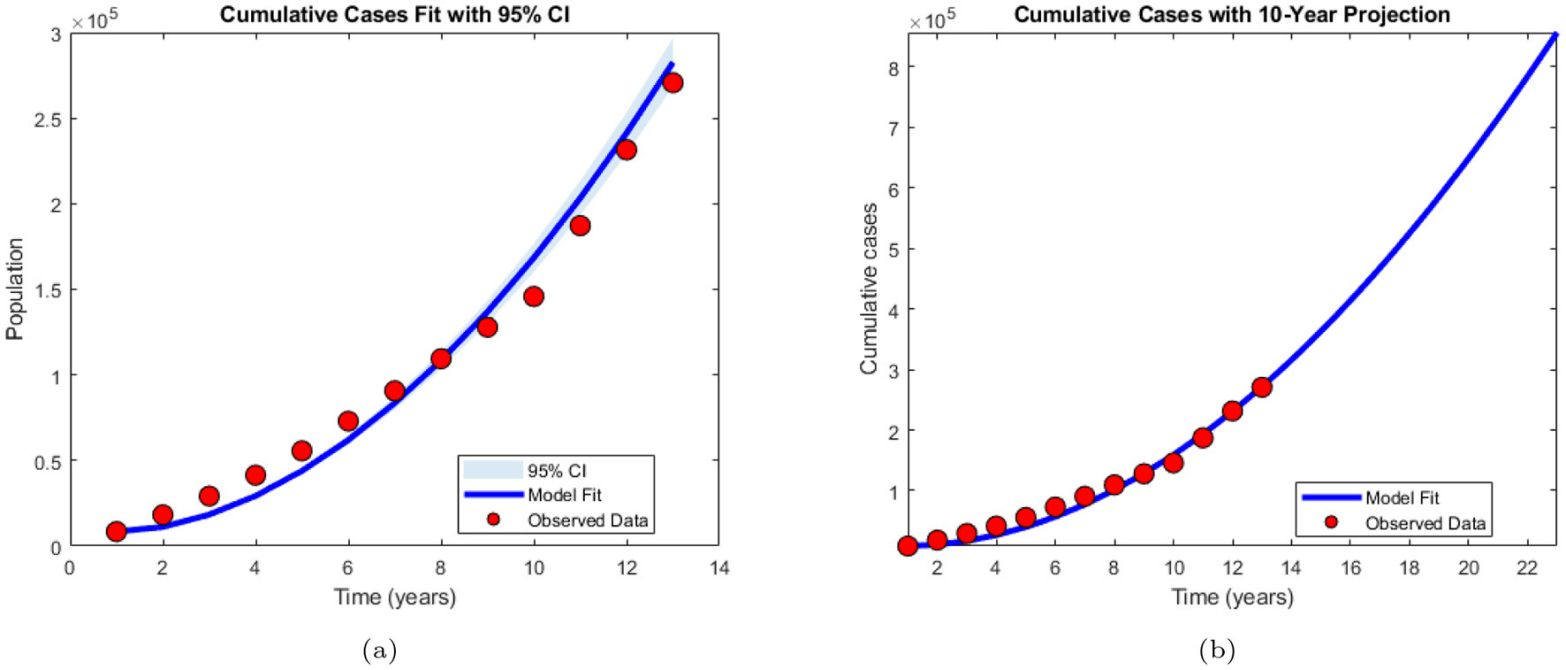
**(a)** Model calibration for using the *fmincon* optimization routine, showing the fitted model output (blue curve) against the observed annual confirmed Syphilis cases in the United States (red dots) from 2011 to 2023, while the light blue patch represents the 95% confidence interval. **(b)** Model fitting with 10-year forecast.

We present the parameters and estimate the model parameters. The natural death rate is obtained from the data on the general life expectancy of MSM in the USA, which is 76.4 years, and hence we estimate the natural death rate, μ for our model to be $\frac{1}{\mu }=\frac{1}{76.4}$ per year (46). We consider recent statistics on the population of MSM in the USA to help estimate Λ. A meta-analysis of five national surveys (2017–2021) reported that approximately 3.3% of U.S. men had sex with another man in the past year, corresponding to an estimated 4.23 × 10^6^ MSM among adult males (47–49). Lifetime prevalence was estimated at 6.2%, indicating that more than one in twenty men in the U.S. have ever had sex with another man during their lifetime (49, 50). From the estimate that 3.3% of U.S. men corresponds to 4.23 × 10^6^ MSM, We approximate the total adult male population as $\frac{4.23\times 10^{6}}{0.033}\approx 1.28\times 10^{8}.$ Applying the 6.2% lifetime prevalence, we obtain 0.062 × 1.28 × 10^8^≈7.9 × 10^6^. Thus, 6.2% corresponds to approximately 7.9 million men in the USA. Therefore, to approximate the recruitment term Λ, we set $\Lambda =\left[\frac{4.23\times 10^{6}}{0.033}-\frac{7.9\times 10^{6}}{76.5}\right]\text{per year}.$ Considering the recovery rate for syphilis, estimates from published modeling studies often assume that with treatment coverage included, models sometimes use γ_*t*_ in the range of [0.1–5]year^−1^, depending on whether treatment coverage and diagnosis delays are explicitly modeled (51, 52). Furthermore, for the rate of waning immunity in syphilis, since recovery does not confer lasting immunity, we set ω to be very large. This aligns with public health guidance and clinical evidence indicating that reinfection is common (53–55). Because recovery from syphilis does not confer protective immunity, individuals are assumed to return immediately to susceptibility following adequate treatment. We set the waning parameter δ to a large value, with a baseline range of δ = [52–365] year^−1^ (53–55). The initial population is $N_{0}=3.0\times 10^{6}$, which is distributed across the subpopulations as follows: *I*_0_ = 500, *L*_0_ = 150, *R*_1, 0_ = 20, and *R*_2, 0_ = 10. The initial number of susceptible individuals is therefore given by *S*_0_ = *N*_0_−(*I*_0_+*L*_0_+*R*_1, 0_+*R*_2, 0_). The initial cumulative number of cases is *C*_0_ = 8482. We used both parameters found in the literature and those we estimate using existing data to fit our model, with results depicted in Figure 6a, while the model parameters are summarized in Table 4.

**Table 4:** Parameter ranges, point values, and references for the syphilis transmission model (normalized per year).

Parameter	Range	Point value	Units	Ref.
Λ	$\left[\frac{4.23\times 10^{6}}{0.033}-\frac{7.9\times 10^{6}}{76.5}\right]$	$\frac{4.23\times 10^{6}}{0.033}$	$\frac{\text{human}}{\text{year}}$	Estimated
μ	-	$\frac{1}{76.4}$	$\frac{1}{\text{year}}$	(46)
β_1_	[0, 1]	8.45372*e*^−7^	$\frac{1}{\text{human}\times \text{year}}$	Fitting
β_2_	[0, 1]	4.58522*e*^−5^	$\frac{1}{\text{human}\times \text{year}}$	Fitting
*u*_1_	[0, 1]	0.004406	$\frac{1}{\text{year}}$	Fitting
*u*_2_	[0, 1]	0.022008	$\frac{1}{\text{year}}$	Fitting
γ_*t*_	-	$\frac{365}{7}$	$\frac{1}{\text{year}}$	(65)
γ_1_	-	$\frac{365}{46}=7.94$	$\frac{1}{\text{year}}$	(17)
γ_2_	[2 − 8]	4	$\frac{1}{\text{year}}$	(7)
δ	[0.2–1]	0.5	$\frac{1}{\text{year}}$	(53–55, 66)
ξ	(0, ∞)	0.24	dimensionless	Assumed
*p*	(0, ∞)	0.3	dimensionless	Varied
*a*	(0, ∞)	0.05	persons^−1^	Assumed
ψ	(0,1)	0.95	dimensionless	Assumed

To calibrate the syphilis transmission model to the observed epidemic data, we performed parameter estimation in MATLAB using the ode45 solver to numerically integrate the system of equations and the fmincon function from the Optimization Toolbox, combined with the MultiStart routine to enhance global convergence and avoid local minima. The estimation aimed to determine the set of parameters that best reproduced the cumulative number of reported infections over time. Four key parameters governing infection transmission and behavioral responses were estimated: the transmission coefficients (β_1_ and β_2_) and the control parameters (*u*_1_ and *u*_2_). These parameters capture the central mechanisms governing transmission, reinfection, and treatment dynamics. All other parameters were fixed at their baseline values specified in Table 4.

The estimation problem was formulated as a nonlinear least-squares optimization. Specifically, the objective function was defined to minimize the discrepancy between the model-predicted and observed cumulative infections, measured as the sum of squared deviations over the observation period:


$$\begin{array}{l} \underset{\theta }{\min}J\left(\theta \right)=||C_{\text{model}}\left(t;\theta \right)-C_{\text{data}}\left(t\right){||}_{2}, \end{array}$$

where ***θ*** = [β_1_, β_2_, *u*_1_, *u*_2_] denotes the parameter vector, *C*_model_(*t*; ***θ***) represents the simulated cumulative infections obtained by integrating the *I*(*t*) trajectory, and *C*_data_(*t*) is the observed cumulative infection data at discrete time points. Parameter bounds were chosen based on biologically and epidemiologically reasonable ranges reported in the literature (see Table 4). The optimization process iteratively adjusted the parameters (***θ*** = β_1_, β_2_, *u*_1_, *u*_2_) until the model closely reproduced the cumulative case data with the smallest possible discrepancy. The resulting parameter estimates, along with their admissible bounds, are summarized in Table 4, and the final calibrated set obtained from this process was used as the baseline for subsequent simulations and scenario analyses conducted under the 95% confidence interval. The quality of the model fit to the observed cumulative Syphilis cases is illustrated in Figure 6a.

Figure 6a shows that the model accurately captures the observed trend in cumulative cases, with the 95% confidence interval (shaded region) remaining narrow, indicating strong reliability of the fitted model. The observed data points (red circles) align closely with the fitted curve (blue line), confirming that the estimated parameters provide a realistic representation of the epidemic dynamics. While in Figure 6b extends the model to generate a 10-year forecast beyond the observed period. The projection reveals a continuous and accelerated increase in cumulative cases, suggesting that without additional control measures or behavioral changes, the total burden of infection is expected to rise substantially over time.

To quantitatively evaluate the goodness of fit, two complementary statistical performance metrics were computed: the root mean square error (RMSE) and the mean absolute percentage error (MAPE). The RMSE measures the overall magnitude of fitting errors in the same units as the data, while the MAPE quantifies the average relative deviation between the model predictions and observations as a percentage. A lower MAPE indicates that the model predictions closely follow the observed data in relative terms, while a smaller RMSE suggests a low overall prediction error. For the present model, the fitting procedure achieved an MAPE of 14.4% and an RMSE of 9376.6, indicating a good overall fit between the model and the observed cumulative infections.

We note that the parameter estimation was performed using a least-squares fitting approach implemented in MATLAB, combining the ode45 solver for numerical integration and fmincon for constrained optimization. Initial conditions were selected based on available epidemiological data and reasonable assumptions consistent with the model structure. To ensure robustness, multiple initial guesses were used in the optimization process, and convergence was assessed based on solver exit flags and residual error minimization. While the model achieves a good fit to observed data, we acknowledge potential identifiability challenges due to parameter correlations, underreporting, and the use of aggregated surveillance data. Therefore, parameter estimates should be interpreted as effective values capturing population-level dynamics rather than precise individual-level quantities.

### 5.2 Global stability analysis

**Figure 7:**
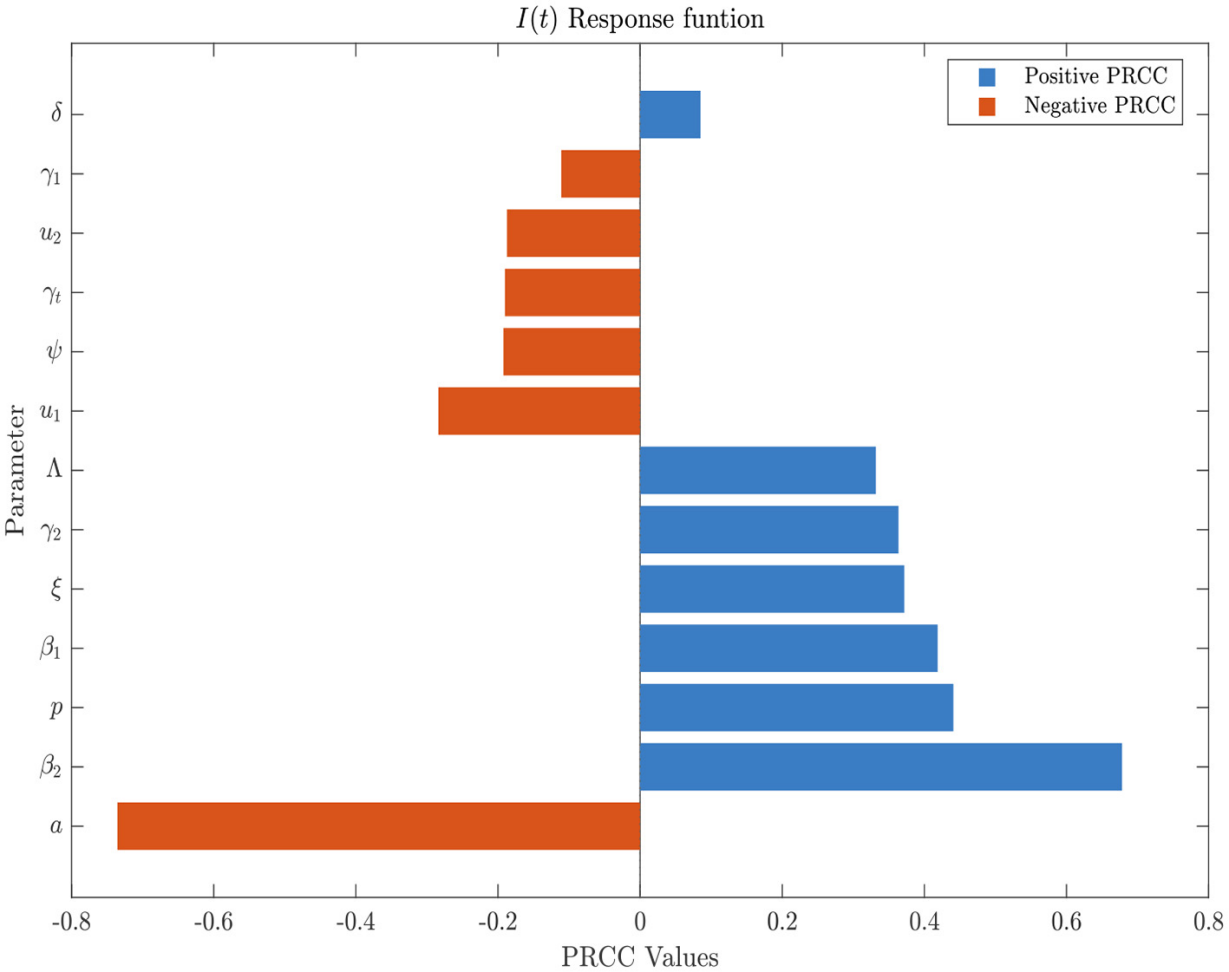
Global sensitivity analysis of model Equation (3) using the infectious class *I*(*t*) as the response function. All parameters were varied while μ was kept constant. A total of 1,200 simulation runs were performed with a step size of 1. The tornado plot illustrates parameters with both positive and negative influences on syphilis dynamics relative to the infectious class.

To determine which parameters most strongly influence syphilis transmission, a global sensitivity analysis (GSA) was carried out using the Latin Hypercube Sampling–Partial Rank Correlation Coefficient (LHS-PRCC) method as described in Marino et al. (56) and Chukwu (57). The infectious population *I*(*t*) was selected as the response variable and summarized by its final value at *t*_max_ = 12 years *Y* = *I*_end_ = *I*(*t*_max_). The natural death rate $\mu =\frac{1}{76.4}\;{\text{yr}}^{-1}$ was fixed at, while the remaining thirteen epidemiological and behavioral parameters were varied within biologically plausible ranges {Λ, β_1_, β_2_, *u*_1_, *u*_2_, γ_*t*_, γ_1_, γ_2_, δ, ξ, *p, a*, ψ}. Parameter samples were generated using Latin Hypercube Sampling *n* = 1200, assuming uniform distributions over the ranges provided in Table 5. For each sampled parameter set, the model was numerically integrated using the ode45 solver in MATLAB with relative and absolute tolerances of 10^−8^ and 10^−10^, respectively. The PRCC between each parameter and the response *Y* was calculated while controlling for the influence of the other parameters. Uncertainty in each PRCC estimate was quantified via 1,200 bootstrap replicates to obtain 95% confidence intervals (CIs). Additionally, two-sided *t*-tests were performed to compute *p*-values for the null hypothesis *H*_0_:ρ_*j*_ = 0. The PRCC values, 95% confidence intervals, and corresponding *p*-values are summarized in Table 5 and depicted in Figure 7.

**Table 5:** LHS and PRCC results for the infectious class response *Y* = *I*_end_ with μ fixed. CI, bootstrap 95% percentile. Sensitive parameters (|PRCC|>0.3, *p* < 0.01) are marked with an asterisk (^*^).

Parameter	Lower	Upper	PRCC	95% CI (Lower, Upper)	*p*-value
*a* ^*^	0.005	0.2	−0.736	[−0.763, −0.706]	5.46 × 10^−203^
$\beta_{2}^{*}$	0	1	0.678	[0.644, 0.715]	5.59 × 10^−161^
*p* ^*^	0	1	0.441	[0.395, 0.489]	1.18 × 10^−57^
$\beta_{1}^{*}$	0	1	0.419	[0.366, 0.468]	1.27 × 10^−51^
ξ^*^	0.1	0.5	0.372	[0.320, 0.422]	3.16 × 10^−40^
$\gamma_{2}^{*}$	2	8	0.363	[0.319, 0.414]	2.05 × 10^−38^
Λ^*^	1.03 × 10^8^	1.54 × 10^8^	0.332	[0.279, 0.381]	6.79 × 10^−32^
*u*_1_	0	1	−0.284	[−0.345, −0.222]	1.87 × 10^−23^
ψ	0.5	0.99	−0.192	[−0.244, −0.137]	2.36 × 10^−11^
γ_*t*_	41.714	62.571	−0.190	[−0.252, −0.129]	3.82 × 10^−11^
*u*_2_	0	1	−0.187	[−0.241, −0.135]	7.71 × 10^−11^
γ_1_	6.348	9.522	−0.111	[−0.172, −0.056]	1.25 × 10^−4^
δ	0.2	1	0.085	[0.029, 0.139]	3.40 × 10^−3^

Parameters identified as sensitive (denoted by an asterisk, ^*^) are those with |PRCC|>0.3 and statistically significant *p*-values (*p* < 0.01). The saturation parameter a^*^ exhibited the strongest (negative) influence on the infectious class (PRCC = −0.736, *p* < 10^−200^), indicating that stronger saturation effects substantially reduce infection levels. Conversely, the reinfection rate $\beta_{2}^{*}$ (PRCC = 0.678) and the primary transmission rate $\beta_{1}^{*}$ (PRCC = 0.419) showed the largest positive effects, confirming that higher transmission and reinfection rates increase infection prevalence. The partner treatment fraction p^*^ (PRCC = 0.441), the latent infectiousness modifier ξ^*^ (PRCC = 0.372), and the progression rate $\gamma_{2}^{*}$ (PRCC = 0.363) also showed significant positive correlations with the infectious class. Similarly, the recruitment rate Λ^*^ (PRCC = 0.332) contributed positively to infection persistence. In contrast, higher treatment rates (*u*_1_) and recovery rates (γ_*t*_, γ_1_) were associated with negative PRCCs, indicating that enhanced treatment and faster recovery reduce the prevalence of infection. Further, the infection dynamics were found to be most sensitive to the transmission parameters (β_1_, β_2_), the non-linear saturation term *a*, and behavioral or progression parameters (*p*, ξ, γ_2_), while demographic and clearance parameters exerted secondary effects. Our analysis provides clear quantitative evidence that the prevalence of infection in the population is primarily governed by transmission and reinfection processes. The parameters β_1_, β_2_, *p*, and ξ collectively regulate the rate of new infections and reinfections, while the saturation parameter *a* modulates how effectively contact rates translate into new cases. In practical terms, this means that interventions aimed at reducing transmission (through partner notification, screening, and safer sexual practices) or limiting reinfection (through improved treatment adherence and follow-up) will have the most substantial impact on controlling syphilis spread. Parameters related to treatment speed and recovery, although less sensitive, still contribute meaningfully to reducing infection persistence. These findings thus highlight the importance of combining behavioral interventions with biomedical control measures to achieve effective and sustained reduction in syphilis transmission. The results from our model sensitivity analysis highlights that transmission and reinfection parameters are the dominant drivers of disease dynamics. This suggests that interventions targeting these mechanisms, such as increased screening, rapid treatment, partner notification, and behavioral risk reduction, are likely to have the greatest impact on reducing disease burden. In particular, the strong influence of the behavioral parameter *a* indicates that public health campaigns promoting awareness and safer practices can substantially reduce transmission. These findings support a combined intervention approach integrating biomedical and behavioral strategies for effective syphilis control.

GSA has also been explored for other epidemic models, see for instance (58–62).

### 5.3 Simulation results varying scenarios

**Figure 8:**
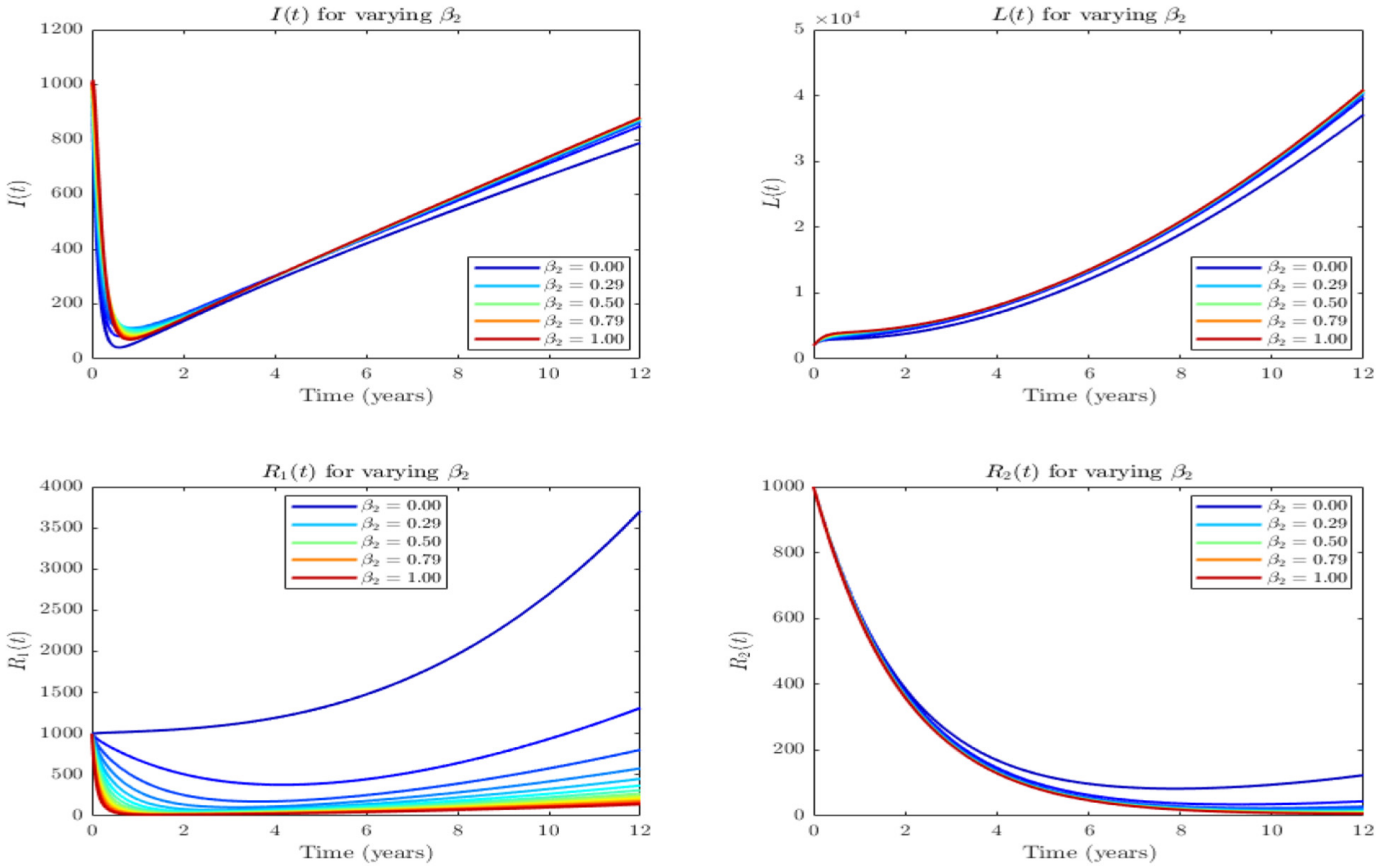
Time evolution of the model compartments for different values of the reinfection rate β_2_, illustrating how repeated infections influence disease persistence. All other parameters are kept fixed as listed in Table 4.

Figure 8 illustrates the temporal evolution of the infected *I*(*t*), latent *L*(*t*), and recovered *R*_1_(*t*) and *R*_2_(*t*) populations under different values of the transmission parameter β_2_, while keeping all other parameters fixed. The panels collectively demonstrate the sensitivity of disease progression to changes in the secondary transmission rate. In the upper left panel, *I*(*t*) initially declines due to the depletion of susceptible individuals, followed by a gradual increase that becomes more pronounced as β_2_ rises. This indicates that higher β_2_ values intensify reinfection or secondary transmission, prolonging the persistence of infection in the population. The corresponding latent population *L*(*t*) (upper–right panel) exhibits a monotonic increase over time, with the growth rate slightly enhanced for larger β_2_, suggesting that elevated secondary transmission facilitates accumulation in the latent stage. The bottom panels show the dynamics of the recovered classes *R*_1_(*t*) and *R*_2_(*t*). Both exhibit a non-monotonic response: for smaller β_2_ values, recovery dominates, leading to a decline after an initial rise; however, as β_2_ increases, reinfection becomes more likely, causing a resurgence in *R*_1_(*t*) and sustained elevation in *R*_2_(*t*). This pattern confirms that a higher β_2_ reduces the effectiveness of recovery in suppressing disease prevalence. Therefore, increasing β_2_ amplifies infection persistence, enlarges the latent reservoir, and weakens long-term recovery effects. These results highlight the critical influence of secondary transmission on disease control efforts and the potential necessity of interventions that specifically reduce β_2_, such as improved treatment follow-up or behavioral change strategies.

**Figure 9:**
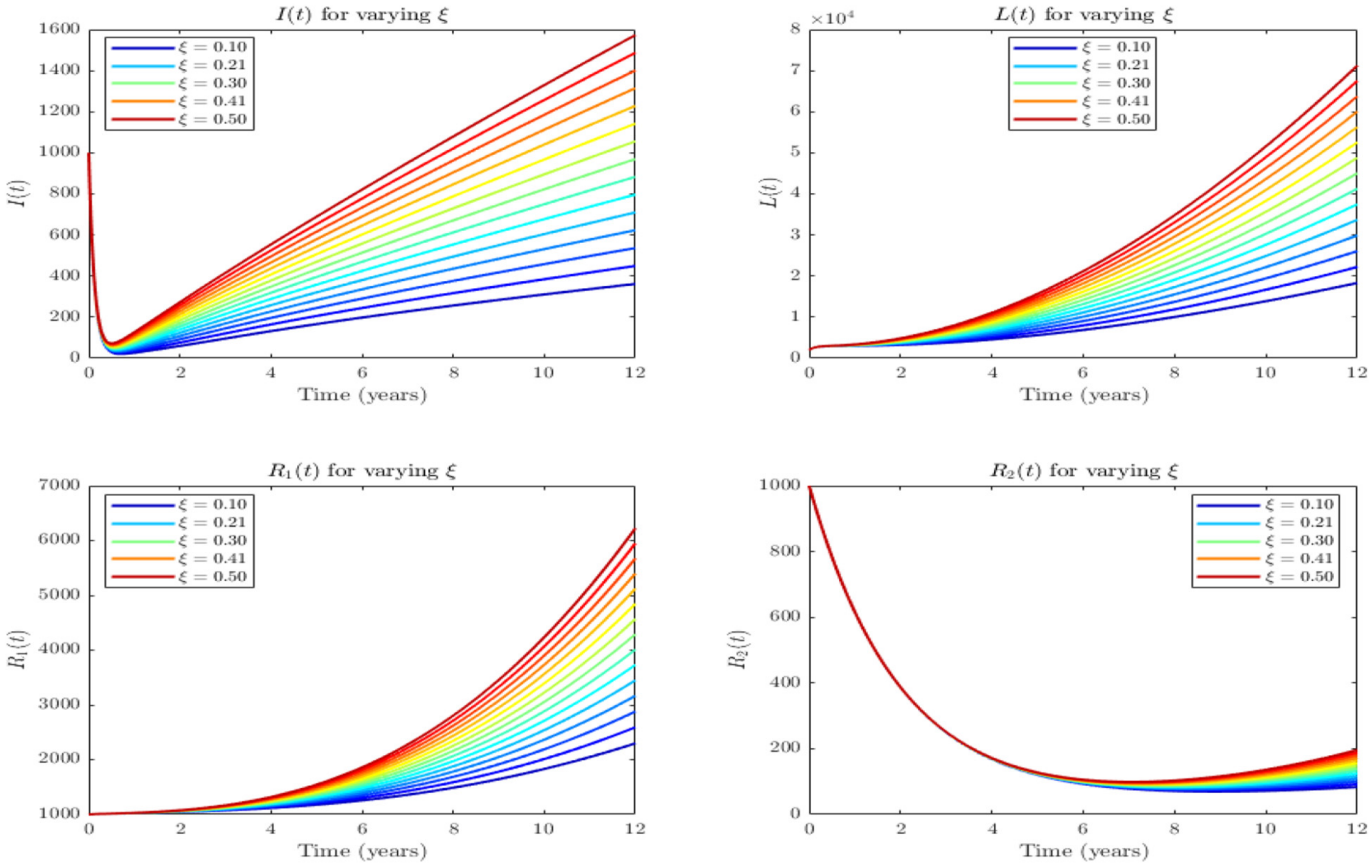
Effect of the relative infectiousness of latent individuals ξ on syphilis transmission dynamics showing the impact of latent-stage transmission on disease persistence. All remaining parameters are kept constant as given in Table 4.

Further, Figure 9 shows the time evolution of the infected *I*(*t*), latent *L*(*t*), and recovered *R*_1_(*t*) and *R*_2_(*t*) populations for different values of the parameter ξ, which represents the progression or reactivation rate from the latent to infectious class. All other parameters are held constant to isolate the influence of ξ on disease transmission dynamics. As observed in the upper-left panel, increasing ξ markedly amplifies the growth of the infectious population *I*(*t*). For small ξ, infection remains at relatively low levels, but higher ξ values cause *I*(*t*) to increase sharply over time, indicating that enhanced reactivation or progression significantly accelerates the spread of infection. The corresponding latent population *L*(*t*) (upper–right panel) also rises monotonically with ξ, suggesting that higher progression rates sustain a larger reservoir of latent individuals that continuously replenish the infectious people. The lower panels depict the evolution of the recovered populations *R*_1_(*t*) and *R*_2_(*t*). Both *R*_1_(*t*) and *R*_2_(*t*) grow faster as ξ increases, reflecting the higher turnover of individuals through infection and recovery pathways. However, the persistence of high *R*_1_(*t*) and the slow decay of *R*_2_(*t*) for large ξ imply that frequent reactivation leads to recurrent infections, thereby enlarging the recovered classes over time. Additionally, the figure shows that increasing ξ enhances disease persistence by accelerating the transition of individuals from the latent to the infectious compartment. This leads to higher peaks of infection, larger latent and recovered populations, and sustained disease transmission. Thus, reducing ξ, for instance, through improved treatment adherence or preventive therapy among latent carriers, could be an effective strategy for mitigating the long-term prevalence of infection.

**Figure 10:**
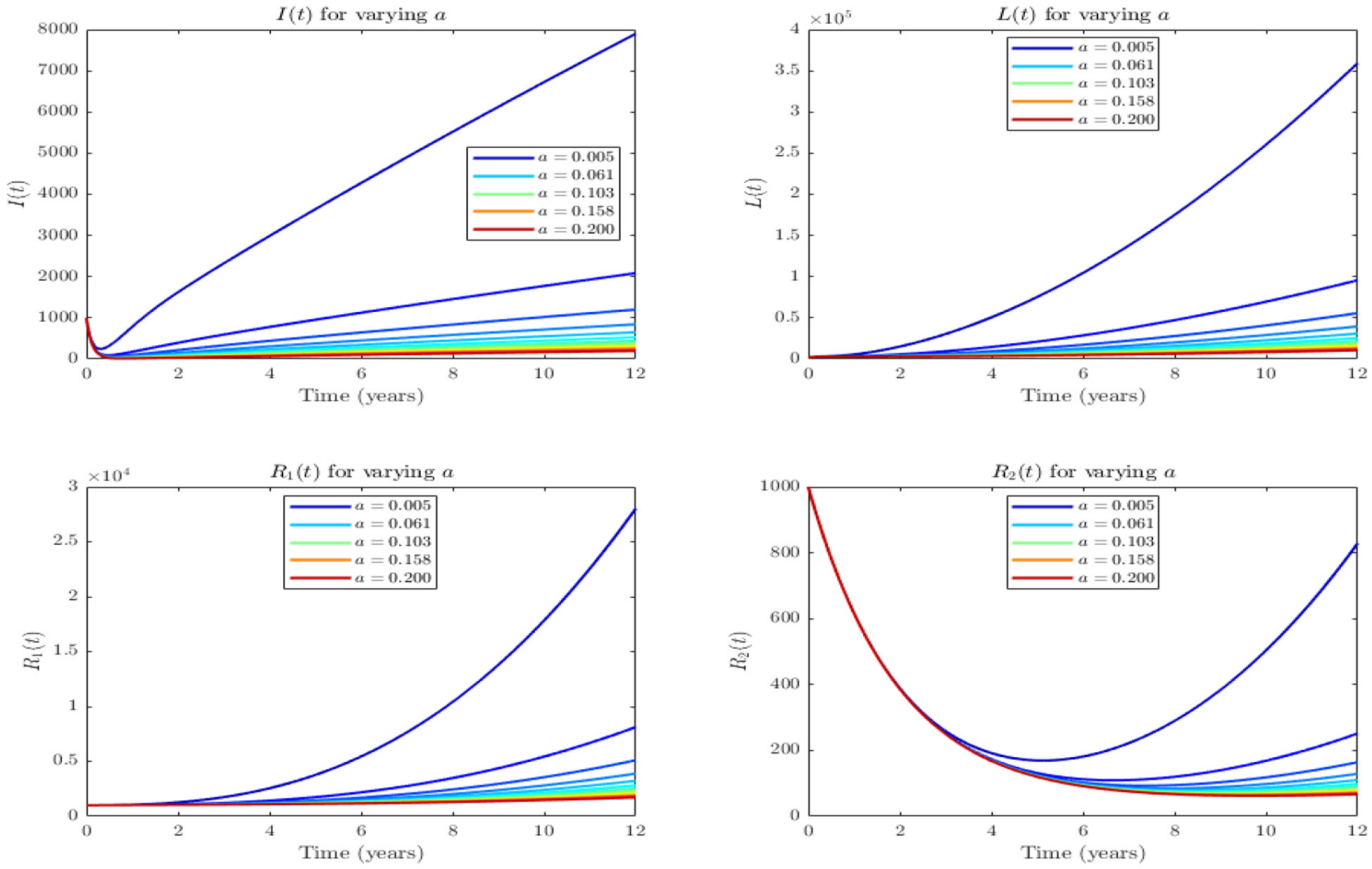
Impact of the behavioral adaptation coefficient *a* on the evolution of the disease compartments demonstrating the effect of adaptive contact reduction on syphilis transmission. All other parameters remain fixed at their baseline values shown in Table 4.

Figure 10 depicts the temporal behavior of the infected *I*(*t*), latent *L*(*t*), and recovered *R*_1_(*t*) and *R*_2_(*t*) populations under different values of the parameter *a*, while keeping all other parameters fixed. The parameter *a* represents the fraction of individuals who avoid progression or reactivation (for instance, due to partial immunity or reduced susceptibility), and thus acts as a moderating factor in the transmission dynamics. The upper-left panel shows that as *a* increases, the number of infected individuals *I*(*t*) rises substantially. Smaller values of *a* suppress infection, resulting in slower growth of *I*(*t*) over time. In contrast, higher *a* values amplify infection prevalence, indicating that greater persistence of infectious individuals (or reduced recovery efficacy) enhances the spread of disease. Similarly, the latent class *L*(*t*) (upper–right panel) grows more rapidly with larger *a*, suggesting that this parameter contributes to maintaining a significant latent reservoir in the population. In the lower panels, both recovered classes, *R*_1_(*t*) and *R*_2_(*t*), display a strong dependence on *a*. Higher *a* values produce a faster rise in *R*_1_(*t*) and *R*_2_(*t*), reflecting the cumulative effect of increased infection and recovery turnover. Conversely, for smaller values of *a*, recovery is less pronounced, and the system tends toward lower equilibrium values of the recovered compartments. Biologically, the figure reveals that increasing *a* promotes infection persistence and enlarges both the latent and recovered populations. This behavior highlights that *a* plays a crucial role in modulating the long-term prevalence of disease. Reducing *a* through enhanced immunity, improved treatment compliance, or targeted behavioral interventions could effectively mitigate the disease burden in the population.

**Figure 11:**
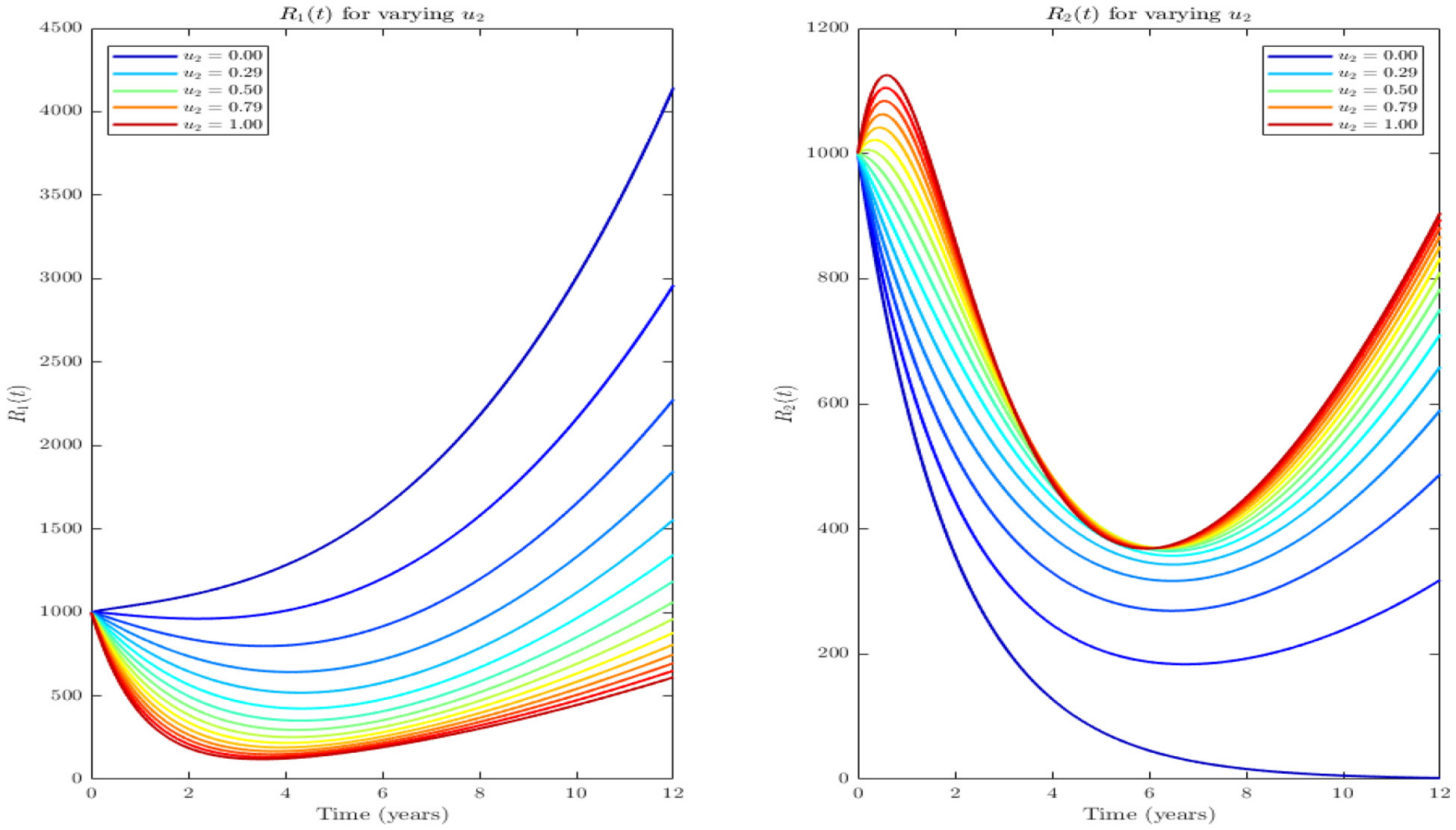
Influence of the recovery-enhancement control *u*_2_ on the distribution of the recovered classes highlighting the influence of improved progression from *R*_1_ to the immune class *R*_2_. All other parameters are held constant at the baseline values specified in Table 4.

Additionally, Figure 11 illustrates the temporal dynamics of the recovered classes *R*_1_(*t*) and *R*_2_(*t*) under different levels of the control variable *u*_2_, which represents the intensity of food recall and control interventions. All other parameters are held fixed to isolate the effect of *u*_2_ on the recovery and reinfection pathways. As shown in the left panel, an increase in *u*_2_ substantially reduces the magnitude of *R*_1_(*t*) in the early stages, indicating that stronger food control measures effectively limit exposure and thereby decrease the number of newly recovered individuals. However, for smaller values of *u*_2_, the curve rises sharply after the initial decline, reflecting a higher reinfection rate due to inadequate intervention coverage. The right panel shows a similar trend for *R*_2_(*t*), where higher *u*_2_ values lead to a faster decline and lower steady-state levels of recovered individuals. This suggests that more effective food safety measures not only prevent primary infection but also minimize reinfection cycles, stabilizing the recovered population over time. Therefore, increasing the control effort *u*_2_ markedly suppresses both infection and reinfection processes by reducing contamination and exposure. These findings underscore the critical role of sustained food recall and control interventions in curbing disease transmission and promoting long-term epidemiological stability.

## 6 Discussions

### 6.1 Conclusion

This study developed and analyzed a compartmental model to understand syphilis transmission among MSM in the United States. The model incorporated reinfection, two-stage treatment, and treatment failure, together with behavioral adaptation and temporary immunity. Analytical results included the derivation of the basic and control reproduction numbers, the stability of the disease-free and endemic equilibria, and the existence of backward bifurcation, which indicates that reducing the reproduction number below one may not guarantee disease elimination. Using U.S. surveillance data from 2011 to 2023, joinpoint regression identified three epidemic inflection points in 2017, 2019, and 2021 corresponding to distinct changes in transmission trends. Model calibration yielded a good fit to the observed data, indicating that reinfection, behavioral response, and incomplete treatment have a significant influence on persistence. Global sensitivity analysis showed that transmission and reinfection parameters, as well as behavioral factors, are the dominant drivers of infection dynamics, while treatment and recovery parameters exert secondary effects. Simulation experiments demonstrated that enhanced screening, effective treatment, and post-exposure prophylaxis can substantially reduce the disease burden among MSM populations. Furthermore, the findings suggest that sustainable syphilis control requires an integrated approach combining biomedical interventions with behavioral and awareness-based strategies.

Beyond biomedical and behavioral interventions, the effectiveness of syphilis control strategies critically depends on the capacity and training of healthcare professionals responsible for implementation. Clinical guidelines, including the administration of BPG and the use of doxy-PEP, require accurate diagnosis, adherence to protocols, and timely delivery of care (63). Emerging evidence indicates that large-scale health education initiatives, particularly those mediated by digital technologies, can significantly improve clinical practice and epidemiological outcomes (5). For example, the Brazilian “Sífilis Não” program has demonstrated that technology-supported professional training enhances surveillance, diagnosis, and treatment adherence, contributing to measurable reductions in disease burden (5, 64). Moreover, studies in high-risk settings, including prison systems, highlight that strengthening healthcare workforce capacity directly influences infection control outcomes (64). These findings underscore that sustainable syphilis control requires not only effective biomedical interventions but also continuous investment in professional training and health education systems. Therefore, integrating mathematical modeling insights with workforce development strategies may enhance the real-world impact of public health policies targeting syphilis and other sexually transmitted infections.

### 6.2 Model limitations

Despite capturing key epidemiological and behavioral mechanisms, the proposed model has several limitations. First, the model assumes homogeneous mixing within the MSM population, thereby overlooking heterogeneity in partnership networks, assortative mixing, and contact concurrency that can strongly influence transmission. In reality, infection risks vary across subgroups defined by age, HIV status, and sexual activity levels, which may affect the estimated reproduction number and persistence thresholds. Second, the model does not incorporate stochastic effects or demographic fluctuations, which can be important when case numbers are low or during the early stages of an outbreak. The deterministic formulation may therefore overestimate the stability of equilibrium states. Third, the model omits spatial variation and mobility patterns, which could help capture city or region-specific trends observed in U.S. surveillance data. Fourth, the two-stage treatment and reinfection dynamics are represented with fixed mean rates, assuming uniform adherence and equal treatment efficacy. In practice, treatment outcomes vary due to differences in access, adherence, and antibiotic response, especially among individuals with repeated infections. The absence of detailed pharmacodynamic or behavioral adherence processes limits the precision of treatment-related predictions. Fifth, the model does not explicitly account for co-infections such as HIV, gonorrhea, or Chlamydia, which can modify syphilis transmission and disease progression. Nor does it incorporate risk compensation behaviors related to PrEP use or post-exposure prophylaxis uptake, which may alter contact rates over time. Sixth, model calibration relied on aggregated national surveillance data from 2011 to 2023, which may mask local variation and underreporting. Incorporating disaggregated, behavioral, and serological data could improve model realism and parameter identifiability in future studies.

Finally, we note that the proposed compartmental framework represents a simplified abstraction of complex real-world transmission processes. In particular, the model assumes homogeneous mixing within the MSM population and does not explicitly capture detailed sexual network structures, partnership concurrency, or assortative mixing patterns. To partially account for behavioral and network heterogeneity, we incorporate aggregated parameters such as effective transmission rates, reinfection rates, and a non-linear behavior-change function that reduces contact rates as prevalence increases. These terms serve as proxies for underlying behavioral adaptation and contact variability observed in real-world populations. Furthermore, clinical variability, including differences in treatment response, adherence, and diagnosis delays, is represented through averaged treatment and progression parameters. While this approach enables analytical tractability and provides population-level insights, it may not fully capture individual-level heterogeneity. We acknowledge that network-based or agent-based models could offer a more detailed representation of these processes. However, the present framework provides a balance between interpretability, analytical insight, and data-driven calibration.

### 6.3 Future work

Future research should extend this framework by incorporating stochastic processes, network-based contact structures, and co-infection dynamics. Linking the model to real-time data streams and employing Bayesian inference or data assimilation techniques could enhance predictive accuracy and enable adaptive, data-driven intervention strategies for MSM populations. Further extensions may include more detailed modeling of treatment adherence, spatial heterogeneity, and behavioral feedback associated with biomedical prevention strategies. In addition, integrating the mechanistic framework with optimization tools could support improved forecasting and intervention planning. Finally, future work will focus on rigorous parameter identifiability analysis using approaches such as profile likelihood, Bayesian methods, or structural identifiability techniques, as well as the use of richer datasets to improve parameter estimation.

## Data Availability

The original contributions presented in the study are included in the article/supplementary material, further inquiries can be directed to the corresponding authors.

## Appendix

### Plot of the behavior change function

Figure A1 illustrates the behavioral response function $f\left(I+\xi L\right)=\frac{1}{1+a\left(I+\xi L\right)}$. As the combined infection measure (*I*+ξ*L*) increases, the function *f*(*I*+ξ*L*) decreases monotonically, indicating a diminishing behavioral response as exposure to infection increases. This inverse non-linear relationship indicates that individuals become progressively less responsive to preventive behavior as the perceived risk of infection increases.
